# TMEM87A suppresses ferroptosis and increases cancer immunotherapy resistance by maintaining the Golgi apparatus pH homeostasis

**DOI:** 10.1038/s43018-026-01156-9

**Published:** 2026-04-21

**Authors:** Jing Li, Yuhan Zhou, Xiong Li, Songlin Yin, Yuan Gao, Haotian Shang, Yongfeng Lai, Liguo Yang, Ying Xue, Xiaoxiao Li, Yan Li, Zhenzhen Chang, Jing Chen, Xiang Cheng, Xiaoyan Zhang, Qian Chu, Fujia Lu, Weimin Wang

**Affiliations:** 1 Department of Immunology, School of Basic Medicine, Tongji Medical College and State Key Laboratory for Diagnosis and Treatment of Severe Zoonotic Infectious Diseases, Huazhong University of Science and Technology, Wuhan, China; 2 Department of Gynecology and Obstetrics, the Central Hospital of Wuhan, Tongji Medical College, Huazhong University of Science and Technology, Wuhan, China; 3 Department of Oncology, Tongji Hospital, Tongji Medical College, Huazhong University of Science and Technology, Wuhan, China; 4 Cancer Center, Union Hospital, Tongji Medical College, Huazhong University of Science and Technology, Wuhan, China; 5 Department of Cardiology, Union Hospital, Tongji Medical College, Huazhong University of Science and Technology, Wuhan, China; 6 College of Biomedicine and Health, College of Life Science and Technology, Huazhong Agricultural University, Wuhan, China; 7 Hubei key Laboratory of Drug Target Research and Pharmacodynamic Evaluation, Wuhan, China; 8 Cell Architecture Research Institute, Huazhong University of Science and Technology, Wuhan, China

**Keywords:** Cancer, Tumour immunology, Cell death, Organelles

## Abstract

Most membrane-bound organelles have been linked to the initiation and execution of ferroptosis. However, the role of the Golgi apparatus and its resident proteins in ferroptosis remain elusive. Here we show that ferroptosis inducer triggers rapid oxidation of Golgi membrane lipids in the early phase of ferroptosis, resulting in disruption of Golgi pH. The Golgi-localized transmembrane protein TMEM87A is identified to mediate ferroptosis resistance through buffering Golgi pH. Depletion of TMEM87A leads to Golgi overacidification, which impairs FSP1-mediated reduction of coenzyme Q. In vivo, TMEM87A ablation suppresses the progression of multiple murine tumors including melanoma, colorectal cancer and liver cancer. TMEM87A ablation also enhances antitumor T cell responses and potentiates PD1 blockade therapy. Clinically, tumoral TMEM87A expression negatively correlates with immunotherapy response and treatment outcome. Our study reveals that TMEM87A functions as a suppressor of tumoral ferroptosis by maintaining Golgi pH homeostasis and targeting TMEM87A is potent to augment cancer immunotherapy.

## Abstract (web summary)

Li et al. show that TMEM87a maintains the Golgi pH homeostasis in response to ferroptosis-induced Golgi lipid peroxidation. TMEM87a ablation sensitizes multiple cancer types to ferroptosis, increasing antitumor immunity and response to immunotherapy.

## Main

Ferroptosis is a form of cell death triggered by excessive accumulation of lipid peroxidation^1,2^. The plasma membrane (PM) is the primary site where polyunsaturated fatty acid (PUFA)-containing phospholipids are peroxidized, leading to membrane rupture and ferroptosis execution^3^. In addition to the PM, multiple subcellular organelle membranes contribute to ferroptosis. Accumulated lipid peroxidation on mitochondrial membrane or increased membrane permeability have been shown to fine-tune the sensitivity of ferroptosis^4,5^. Recently, the endoplasmic reticulum (ER) membrane was identified as the initial site where lipid peroxidation occurs before the PM^6,7^. Intriguingly, the Golgi apparatus, a membranous-bound organelle responsible for processing and sorting proteins and lipids, is involved in regulation of oxidative stress and exhibits membrane lipid peroxidation^8–10^. However, how the Golgi apparatus responds to ferroptosis induction and whether Golgi-localized proteins can regulate ferroptosis execution remains unclear.

As a tumor-suppressive mechanism, ferroptosis can be triggered without synthetic chemical inducers, such as the deprivation of cystine^11,12^ or methionine^13^ or the supplementation of PC-PUFA_2_ lipids^14^. Interestingly, CD8^+^ T cells have been shown to promote ferroptosis of tumor cells through inhibition of cystine uptake^15^ or elevation of arachidonic acid metabolism^16^. Multiple attempts by combining immune checkpoint blockade (ICB) with different ferroptosis inducers have indicated the synergistic antitumor efficacy across various cancer models^17,18^. Therefore, targeting ferroptosis can not only directly eliminate tumor cells but also overcome immunotherapy resistance.

TMEM87A is an evolutionarily conserved transmembrane protein that localizes predominantly to the Golgi apparatus and participates in retrograde trafficking^19^. It contains a Golgi dynamics domain and seven transmembrane helix domains^20,21^. Recently, TMEM87A was identified as a voltage-dependent cation channel that contributes to Golgi pH homeostasis in human astrocytes, with its deficiency leading to hippocampal-dependent memory deficits in mice^21^. Additionally, TMEM87A is found to locate on PM of melanoma cells to support the mechanoelectrical transduction^22^ and has been characterized as an ion channel expressed in sensory neurons to mediate light touch transduction^23^. However, the roles of TMEM87A in cell death and cancer immunity have not been studied.

Here we find that lipid peroxidation accumulated on Golgi membrane causes the elevation of Golgi pH, which in turn protects tumor cells from ferroptosis execution. Golgi-localized TMEM87A can regulate ferroptosis by buffering Golgi pH. In vivo, TMEM87A loss promotes tumoral ferroptosis, potentiates antitumor T cell responses and synergizes with ICB therapy. Collectively, our study unveils the functions of TMEM87A on ferroptosis regulation and cancer immunity.

## Results

### Golgi pH is disturbed in the early phase of ferroptosis

**Fig. 1:**
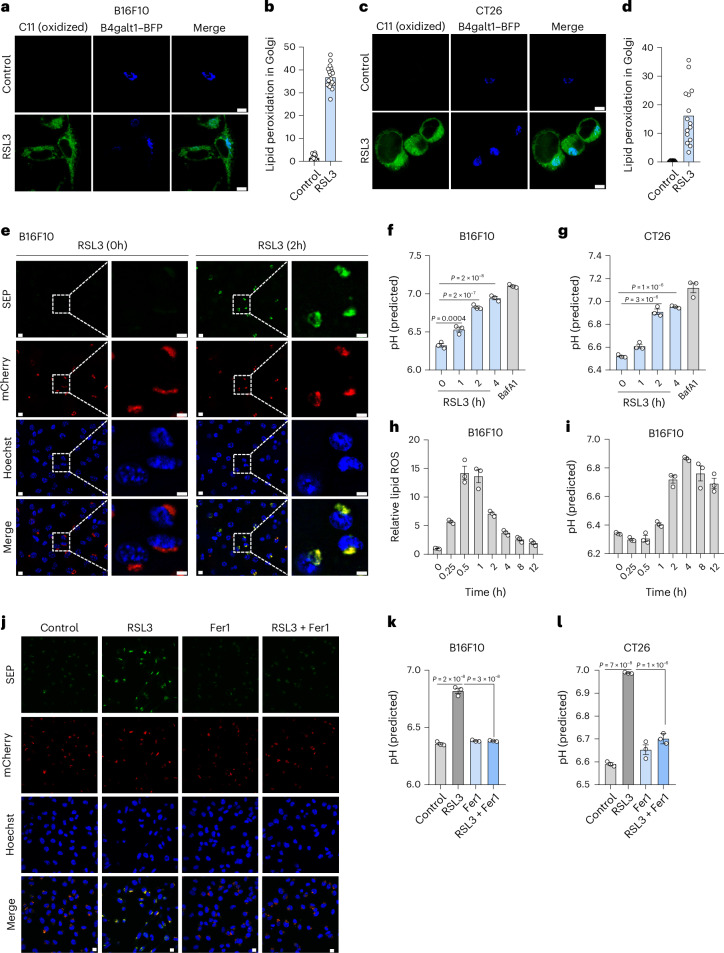
The pH of the Golgi apparatus is disturbed upon ferroptosis induction. **a**–**d**, Representative images of lipid peroxidation stained by BODIPY C11 and Golgi marked by B4galt1–BFP in B16F10 cells (**a**) and CT26 cells (**c**) treated with 2 μM RSL3 for 30 min (B16F10) or 2 h (CT26). Scale bar, 10 μm. Quantification of mean fluorescence intensity of oxidized BODIPY C11 (green) in Golgi (BFP^+^ (blue) area) (**b**,**d**) to indicate Golgi lipid peroxidation (**b**, *n* = 19 (control) or 20 cells (RSL3); **d**, *n* = 15 cells (control, RSL3)). The data shown are representative results from three independent experiments with similar results. **e**, Representative images of B4galt1–mCherry–SEP expressed in B16F10 cells treated by RSL3 (2 μM) for 0 or 2 h, shown in the same field of view. Scale bar, 10 μm (left) or 5 μm (right). **f**,**g**, Quantification of the Golgi pH in B16F10 (**f**) or CT26 (**g**) cells treated with 2 μM RSL3 for indicated time. BafA1 treatment (0.2 μM) serves as a positive control (*n* = 3 biological replicates). The pH was predicted with the calibration curve in Extended Data Fig. 1i-k. **h**,**i**, Changes in relative lipid peroxidation (**h**) and predicted Golgi pH (**i**) in B16F10 cells during treatment with RSL3 (2 μM) for the indicated times (*n* = 3 biological replicates). **j**, Representative images of B4galt1–mCherry–SEP fluorescence signals in B16F10 cells treated with RSL3 (2 μM) for 2 h with or without Fer1 (10 μM). Scale bar, 10 μm. **k**,**l**, Quantification of the Golgi pH in B16F10 (**k**) or CT26 cells (**l**) treated with 2 μM RSL3 with or without Fer1 (10 μM) (*n* = 3 biological replicates). Data shown are the mean (**b**,**d**) or mean ± s.e.m. (**f**–**i**,**k**,**l**). *P* values are indicated. Statistical analyses were conducted using a one-way ANOVA (**f**,**g**,**k**,**l**). Source data

**Extended Data Fig. 1:**
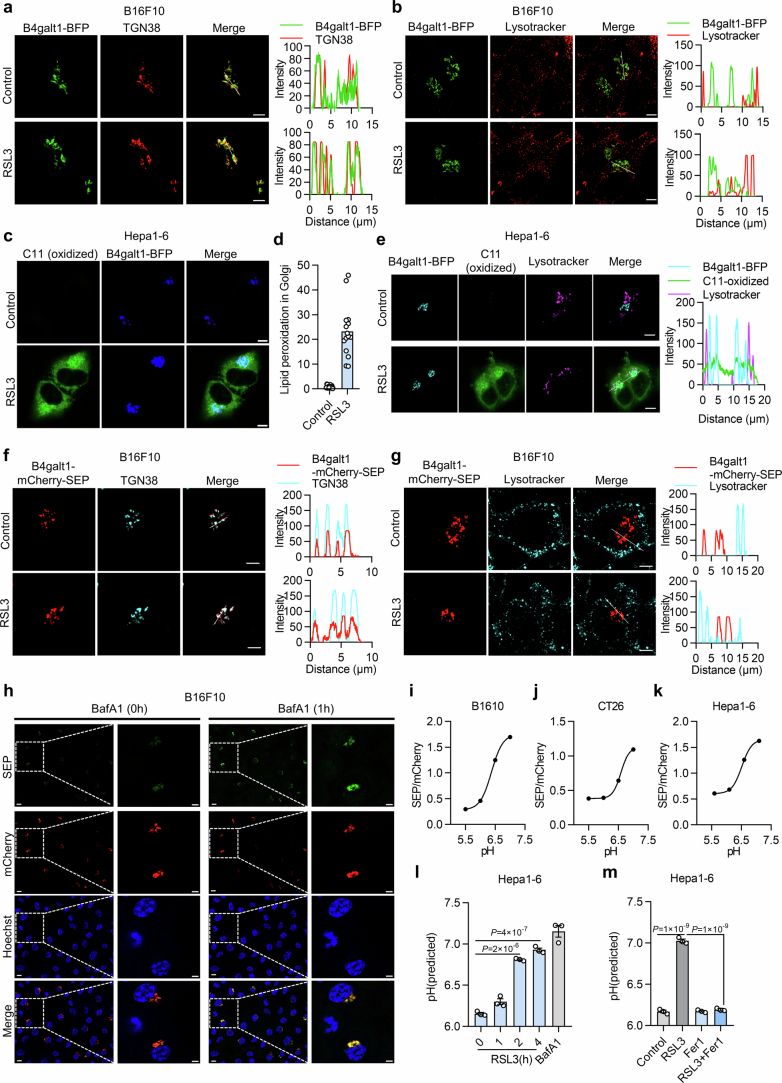
Golgi pH is elevated by ferroptotic lipid peroxidation. **a**, Representative images of B4galt1-BFP (Green) and TGN38 (Red) labelled Golgi in B16F10 cells treated with RSL3 (2 μM) for 2 h, as assessed by confocal microscopy (left). Line scans quantifying the fluorescence intensities of B4galt1-BFP and TGN38 (right). Scale bar, 5 μm. **b**, Representative images of B4galt1-BFP (Green) and Lysotracker (Red) stained lysosomes in B16F10 cells treated with RSL3 (2 μM) for 2 h, as assessed by confocal microscopy (left). Line scans quantifying the fluorescence intensities of B4galt1-BFP and Lysotracker (right). Scale bar, 5 μm. **c, d**, Representative images of lipid peroxidation stained by BODIPY C11 and Golgi marked by B4galt1-BFP in Hepa1-6 cells (**c**) treated with 1 μM RSL3 for 2 h. Scale bar, 10 μm. Quantification of the mean fluorescence intensity of oxidized BODIPY C11 (Green) in Golgi (BFP^+^ (Blue) area) to indicate Golgi lipid peroxidation (**d**). n = 17 cells (Control) or 15 cells (RSL3). The data were shown as representative results from three independent experiments with similar results. **e**, Representative images of lipid peroxidation stained by BODIPY C11 (Green for oxidized BODIPY C11), B4galt1-BFP (Cyan) labelled Golgi and Lysotracker (Magenta) stained lysosomes in Hepa1-6 cells treated with RSL3 (left). Line scans quantifying their fluorescence intensities (right). Scale bar, 5 μm. **f**, Representative images of B4galt1-mCherry-SEP (Red) and TGN38 (Cyan) labelled Golgi in B16F10 cells treated with RSL3 (2 μM) for 2 h (left). Line scans quantifying their fluorescence intensities (right). Scale bar, 5 μm. **g**, Representative images of B4galt1-mCherry-SEP (Red) and Lysotracker (Cyan) labelled lysosomes in B16F10 cells treated with RSL3 (2 μM) for 2 h (left). Line scans quantifying their fluorescence intensities (right). Scale bar, 5 μm. **h**, Representative images of B4galt1-mCherry-SEP fluorescent signals in B16F10 cells upon BafA1 treatment (1 μM) for 0 or 1 h, shown in the same field of view. Scale bar, 10 μm (left) or 5 μm (right). The data were shown as representative results from three independent experiments with similar results. **i- k**, Calibration curve of SEP/mCherry fluorescence ratios correlated with intracellular Golgi pH in Golgi pH sensor expressing cells of B16F10 (**i**), CT26 (**j**) and Hepa1-6 (**k**). **l**, Quantification of the Golgi pH in Hepa1-6 cells treated with 2 μM RSL3 for indicated time. BafA1 treatment (0.2 μM) serves as a positive control (n = 3 biological replicates). **m**, Quantification of the Golgi pH in Hepa1-6 cells treated with 2 μM RSL3 with or without 10 μM Fer1 (n = 3 biological replicates). Data were shown as mean (**d**) or mean ± SEM (**l**, **m**). *P* values were indicated. Statistical analyses were conducted by one-way ANOVA in (**l**, **m**). Source data

Given the proximity of the Golgi apparatus to the peroxidation-prone ER^6^, we speculated that the Golgi membrane might also accumulate lipid peroxides during ferroptosis. To monitor this, we labeled Golgi with blue fluorescent protein (BFP) fused to the Golgi-targeting sequence of β-1,4-galactosyltransferase 1 (B4galt1). B4galt1–BFP colocalized with the Golgi marker Tgoln1 (TGN38) and this colocalization remained unchanged upon RSL3 treatment (Extended Data Fig. 1a). It did not colocalize with Lysotracker-stained lysosomes (Extended Data Fig. 1b). Using the lipid peroxidation sensor BODIPY C11, we observed rapid accumulation of its oxidation signal on BFP^+^ Golgi membranes within 30 min after RSL3 treatment in murine B16F10 cells (Fig. 1a,b). Similar accumulations of oxidized BODIPY C11 were observed on BFP^+^ Golgi in mouse CT26 (Fig. 1c,d) and Hepa1-6 cells (Extended Data Fig. 1c,d) during the early phase of RSL3-induced ferroptosis and this signal was distinct from lysosomal staining (Extended Data Fig. 1e).

The Golgi apparatus maintains a unique pH environment. To investigate whether Golgi pH is perturbed by lipid peroxidation, we constructed a Golgi-targeted ratiometric pH sensor by fusing the pH-sensitive superecliptic pHluorin (SEP) and pH-insensitive mCherry to B4galt1. We again confirmed that this pH sensor localized to the TGN38^+^ Golgi apparatus (Extended Data Fig. 1f) but not to Lysotracker-stained lysosomes (Extended Data Fig. 1g) and its localization remained unchanged during the early phase of ferroptosis. In B16F10 cells expressing this sensor, the fluorescence of SEP was dramatically increased after 2 h of RSL3 treatment (Fig. 1e). Bafilomycin A1 (BafA1), a vacuolar adenosine triphosphatase (V-ATPase) inhibitor^24^, could also increase the brightness of SEP (Extended Data Fig. 1h). By quantifying the ratio of SEP to mCherry for pH calculation (Extended Data Fig. 1i–k), we found that the Golgi pH increased rapidly and continuously within 4 h of RSL3 treatment (Fig. 1f). The elevation of Golgi pH was also observed in RSL3-treated or BafA1-treated CT26 (Fig. 1g) or Hepa1-6 (Extended Data Fig. 1l) cells.

As lipid peroxides rapidly accumulate on the Golgi membrane upon ferroptosis induction, we sought to determine the temporal relationship between lipid peroxidation and Golgi pH elevation. Following RSL3 treatment, lipid peroxidation rose rapidly and peaked 1 h later (Fig. 1h), whereas the Golgi pH started to rise 1 h later and stayed high until 12 h (Fig. 1i). Strikingly, pretreatment with a radical-trapping antioxidant ferrostatin 1 (Fer1) abolished RSL3-induced enhancement of SEP signal (Fig. 1j) and the elevation of Golgi pH (Fig. 1k,l and Extended Data Fig. 1m). Therefore, we conclude that the Golgi pH elevation is caused by lipid peroxides accumulated on the Golgi membrane upon ferroptosis induction.

### Golgi-resident TMEM87A regulates ferroptosis through buffering the Golgi pH

**Extended Data Fig. 2:**
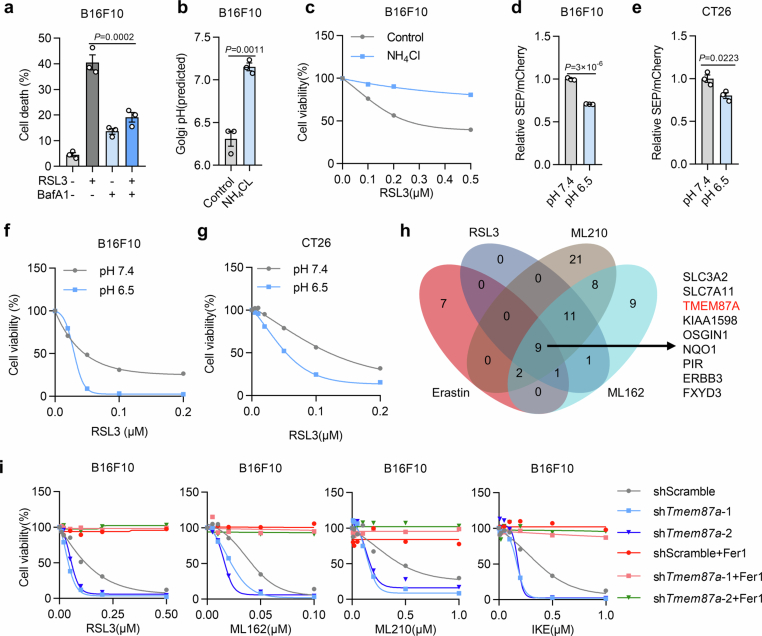
Elevation of Golgi pH protects tumor cells from ferroptosis and knockdown of TMEM87A promotes ferroptosis. **a**, Cell death of B16F10 cells treated with BafA1 (0.2 μM) and RSL3 (0.5 μM). n = 3 biological replicates. **b**, Quantification of the Golgi pH in B16F10 cells treated with NH4Cl (10 mM, 1 h). n = 3 biological replicates. **c**, Relative cell viability of B16F10 cells treated with NH_4_Cl (10 mM) and RSL3. n = 4 technical replicates. **d, e**, Relative SEP/mCherry of B16F10 cells (**d**) or CT26 cells (**e**) cultured in pH 7.4 or pH 6.5 medium. n = 3 biological replicates. **f, g**, Relative cell viability of B16F10 cells (n = 4 technical replicates per group, except for pH 6.5 RSL3 (0.02 μM) group, where n = 3 technical replicates) (**f**) or CT26 cells (n = 3 technical replicates) (**g**) cultured in pH 7.4 or pH 6.5 medium and treated with RSL3. **h**, Venn diagram showing common genes whose expressions were negatively associated with cell sensitivity to RSL3, ML210, ML162 and Erastin. **i**, Relative viabilities of B16F10 cells expressing Scramble or Tmem87a-targeting shRNA in response to RSL3, ML162, ML210 or IKE in the presence of ferrostatin-1 (Fer1, 5 μM). (n = 4 technical replicates per group, except for shScramble ML162 (0.01 μM), sh*Tmem87a*-2 ML210 (0.05, 0.1, 0.2, 0.5, 1 μM) and shScramble + Fer1 IKE (0.2 μM) groups, where n = 3 technical replicates). Data were shown as mean (**c**, **f**, **g**, **i**) or mean ± SEM (**a**, **b**, **d**, **e**). *P* values were indicated. Statistical analyses were conducted by one-way ANOVA in (**a**), two-tailed t-test in (**b**, **d** and **e**). Source data

We next wondered whether this early event of Golgi pH elevation would affect ferroptosis execution. Elevating Golgi pH with BafA1 suppressed RSL3-induced ferroptosis in B16F10 cells (Extended Data Fig. 2a). NH_4_Cl, another Golgi alkalinizing agent^25^, could also increase Golgi pH and inhibit RSL3-induced ferroptosis (Extended Data Fig. 2b,c). We then tried to lower the Golgi pH by incubating cells in an acidic culture medium (pH 6.5) (Extended Data Fig. 2d,e) and found that both B16F10 and CT26 cells became more sensitive to RSL3 (Extended Data Fig. 2f,g). These data suggest that the elevation of Golgi pH may regulate the susceptibility of tumor cells to ferroptosis execution.

**Fig. 2:**
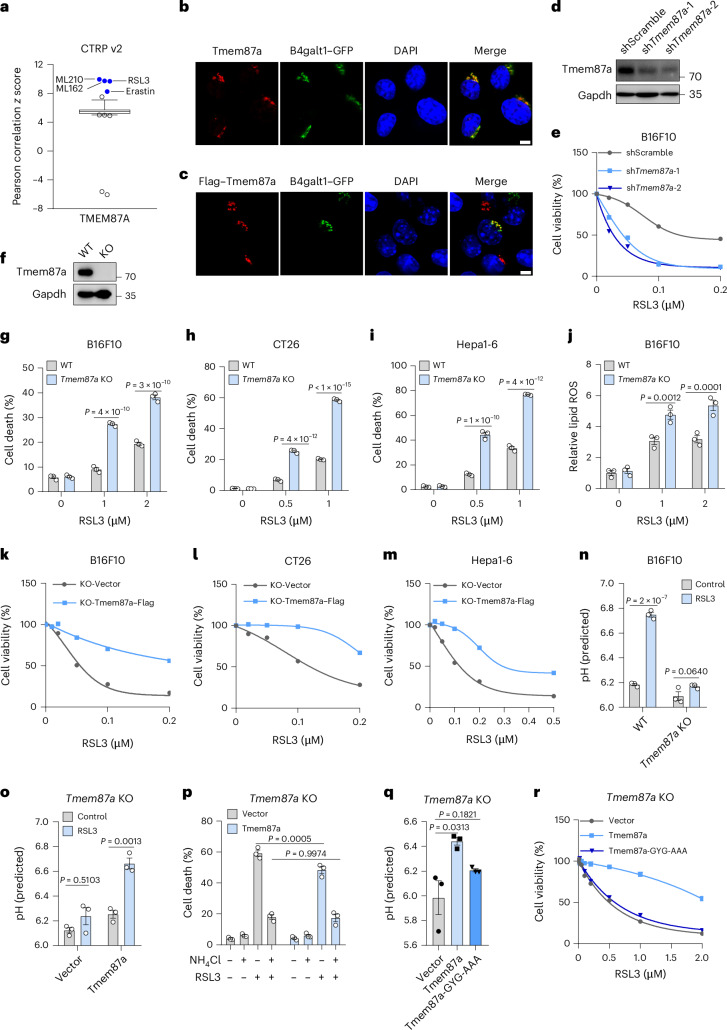
Golgi-localized TMEM87A deficiency increases ferroptosis sensitivity of tumor cells through disrupting Golgi pH. **a**, A high level of TMEM87A expression was correlated with resistance to ferroptosis inducers RSL3, ML162, ML210 and erastin. Data were sourced and analyzed from the CTRP database. Plotted values are Pearson’s correlation coefficients with the minima and maxima of the distributions (line, median; box, 25th and 75th percentiles; whiskers, 10th and 90th percentiles); individual points represent outliers. **b**,**c**, Representative images of colocalization of endogenous Tmem87a (**b**) or ectopically expressed Tmem87a–Flag (**c**) and Golgi marked by B4galt1–GFP in B16F10 cells. Scale bar, 10 μm. **d**, Immunoblot of Tmem87a in B16F10 cells expressing shScramble or sh*Tmem87a*. Gapdh served as a loading control. **e**, Relative cell viability of B16F10 cells expressing shScramble or sh*Tmem87a* treated with RSL3 (*n* = 4 technical replicates, except for the condition of sh*Tmem87a*-2 RSL3 (0.2 µM), where *n* = 3 technical replicates). **f**, Immunoblot of Tmem87a in WT and *Tmem87a*-KO B16F10 cells. Gapdh served as a loading control. **g–i**, Cell death of WT and *Tmem87a*-KO B16F10 cells (**g**), CT26 cells (**h**) and Hepa1-6 cells (**i**) treated with RSL3 (*n* = 3 biological replicates). **j**, Relative lipid ROS of WT and *Tmem87a*-KO B16F10 cells treated with RSL3 (*n* = 3 biological replicates). **k**–**m**, Relative cell viability of empty vector or Tmem87a–Flag-reexpressing *Tmem87a*-KO B16F10 cells (**k**), CT26 cells (**l**) and Hepa1-6 (**m**) cells treated with RSL3 (*n* = 4 technical replicates, except for the condition of CT26 KO-Vector RSL3 (0.05 µM), where *n* = 3 technical replicates). **n**, Quantification of the Golgi pH in WT and *Tmem87a*-KO B16F10 cells treated with RSL3 (2 μM, 2 h) (*n* = 3 biological replicates). **o**, Quantification of the Golgi pH in empty vector or Tmem87a–Flag-reexpressing *Tmem87a*-KO B16F10 cells treated with RSL3 (2 μM, 2 h) (*n* = 3 biological replicates). **p**, Cell death of empty vector or Tmem87a–Flag-reexpressing *Tmem87a*-KO B16F10 cells treated with NH_4_Cl (10 mM) and RSL3 (0.5 μM) (*n* = 3 biological replicates). **q**, Quantification of the Golgi pH in empty vector, Tmem87a-reexpressing or Tmem87a-GYG-AAA mutant-reexpressing *Tmem87a*-KO B16F10 cells (*n* = 3 biological replicates). **r**, Relative cell viability of empty vector, Tmem87a-reexepressing or Tmem87a-GYG-AAA mutant-reexpressing *Tmem87a*-KO B16F10 cells treated with RSL3 (*n* = 4 technical replicates). Data shown are the mean (**e**,**k**–**m**,**r**) or mean ± s.e.m. (**g**–**j**,**n**–**q**). *P* values are indicated. Statistical analyses were conducted using a one-way ANOVA (**q**) or two-way ANOVA (**g**–**j**,**n**–**p**). Source data

**Extended Data Fig. 3:**
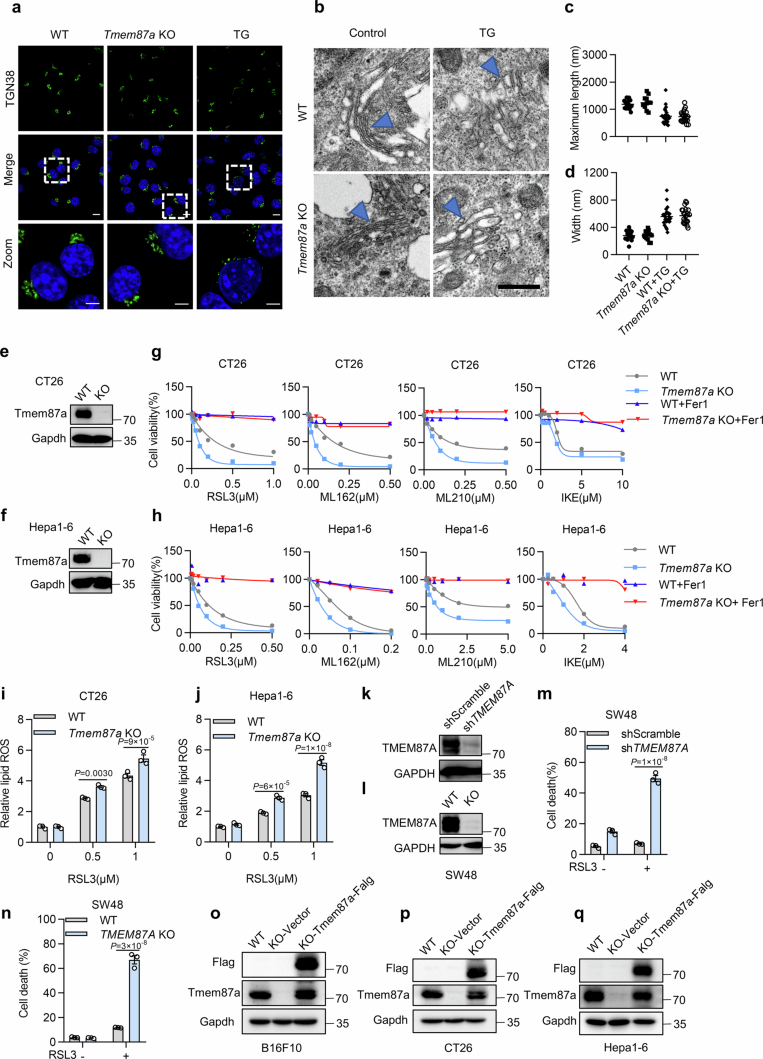
TMEM87A loss promotes ferroptosis of murine and human tumor cells. **a**, Representative images of TGN38 in WT and *Tmem87a* KO B16F10 cells, with thapsigargin (TG) treatment as a positive control. Scale bar, 10 μm (up) or 5 μm (down). **b- d**, TEM images of Golgi in WT and *Tmem87a* KO B16F10 cells, with TG treatment as a positive control (**b**). Blue arrows indicate Golgi. Scale bar, 500 nm. Quantification of maximum length (**c**) and width of Golgi (**d**). n = 16 in WT, 12 in *Tmem87a* KO, 28 in WT + TG, 23 in *Tmem87a* KO group. **e, f**, immunoblot of Tmem87a in WT and *Tmem87a* KO CT26 (**e**) and Hepa1-6 (**f**) cells. **g, h**, Relative viabilities of WT or *Tmem87a* KO CT26 (**g**) and Hepa1-6 (**h**) cells in response to RSL3, ML162, ML210 or IKE in the presence of Fer1. n = 4 technical replicates per group, except for CT26 *Tmem87a* KO + Fer1 ML210 (0.05, 0.2 μM), CT26 WT IKE (2 μM), Hepa1-6 WT RSL3 (0.02, 0.05, 1 μM), Hepa1-6 WT+ Fer1 RSL3 (0.005 μM) and Hepa1-6 WT+ Fer1 IKE (1, 2, 4 μM) groups, where n = 3 technical replicates. **i, j**, Relative lipid ROS of WT and *Tmem87a* KO CT26 (**i**) or Hepa1-6 (**j**) cells in response to RSL3. n = 3 biological replicates. **k,l**, Immunoblot of TMEM87A in SW48 cells expressing Scramble or TMEM87A-targeting shRNA (**k**) or WT and TMEM87a KO SW48 cells (**l**). **m, n**, Cell death of SW48 cells expressing Scramble or TMEM87A-targeting shRNA (**m**) or WT and *TMEM87A* KO SW48 cells (**n**) in response to RSL3. n = 3 biological replicates. GAPDH serves as loading control. **o- q**, Immunoblot of Tmem87a and Flag in WT, an empty vector or Tmem87a-Flag re-expressing *Tmem87a* KO B16F10 (**o**), CT26 (**p**) or Hepa1-6 cells (**q**). Gapdh serves as loading control. Data were shown as mean (**c**, **d**, **g**, **h**) or mean ± SEM (**i**, **j**, **m**, **n**). *P* values were indicated. Statistical analyses were conducted by two-way ANOVA in (**i**, **j**, **m**, **n**). Source data

The Golgi pH homeostasis is maintained by Golgi-resident ion transporter systems^25^. We reasoned that some Golgi-localized proteins might regulate Golgi pH and ferroptosis. The Cancer Therapeutics Response Portal (CTRP) reports correlations between gene expression and cell sensitivity to several classical ferroptosis inducers^26^. Among genes whose higher expression correlates with resistance to RSL3, ML210, ML162 and erastin (Fig. 2a and Extended Data Fig. 2h), only TMEM87A was primarily localized to the Golgi and has the potential to regulate Golgi pH^21^. Thus, we selected TMEM87A for further investigation. We first confirmed that both endogenous Tmem87a and ectopically expressed Tmem87a–Flag were located on B4galt1–GFP-labeled Golgi (Fig. 2b,c). We then explored the effect of Tmem87a on ferroptosis execution. Knockdown of Tmem87a with short hairpin RNA (shRNA) rendered B16F10 cells susceptible to the cytotoxicity induced by RSL3, ML210, ML162 or imidazole ketone erastin (IKE), which could be fully rescued by Fer1 (Fig. 2d,e and Extended Data Fig. 2i). We then ablated Tmem87a with CRISPR–Cas9 editing and confirmed its loss by immunoblot (Fig. 2f). Although Tmem87a-deficient hippocampal astrocytes and neurons were reported to have more disrupted Golgi stacks^21^, we did not observe significant differences in structural integrity and dimensions of Golgi between wild-type (WT) and *Tmem87a*-knockout (KO) tumor cells (Extended Data Fig. 3a–d). We then treated these cells with RSL3 and observed increased cell death in KO cells compared to WT cells (Fig. 2g). Similarly, Tmem87a-deficient CT26 (Extended Data Fig. 3e) and Hepa1-6 (Extended Data Fig. 3f) cells also became more sensitive to RSL3, as revealed by enhanced cell death (Fig. 2h,i) and reduced cell viability (Extended Data Fig. 3g,h). Consistent with this, RSL3 treatment triggered a greater increase in lipid peroxidation in these KO cells than in WT cells (Fig. 2j and Extended Data Fig. 3i,j). *Tmem87a*-KO cells were also more sensitive to ferroptosis induced by ML162, ML210 and IKE and these phenotypes could all be rescued by Fer1 (Extended Data Fig. 3g,h). In addition, both *TMEM87A* knockdown and ablation in human colon cancer SW48 cells notably enhanced RSL3-induced ferroptosis (Extended Data Fig. 3k–n). To further confirm that the ferroptosis sensitization in *Tmem87a*-KO cells was specifically because of the absence of Tmem87a, we reexpressed Tmem87a–Flag in *Tmem87a*-KO B16F10, CT26 or Hepa1-6 cells (Extended Data Fig. 3o–q) and found that their resistance to RSL3 was largely restored (Fig. 2k–m). Therefore, we conclude that TMEM87A deficiency sensitizes both human and murine tumor cells to ferroptosis.

**Extended Data Fig. 4:**
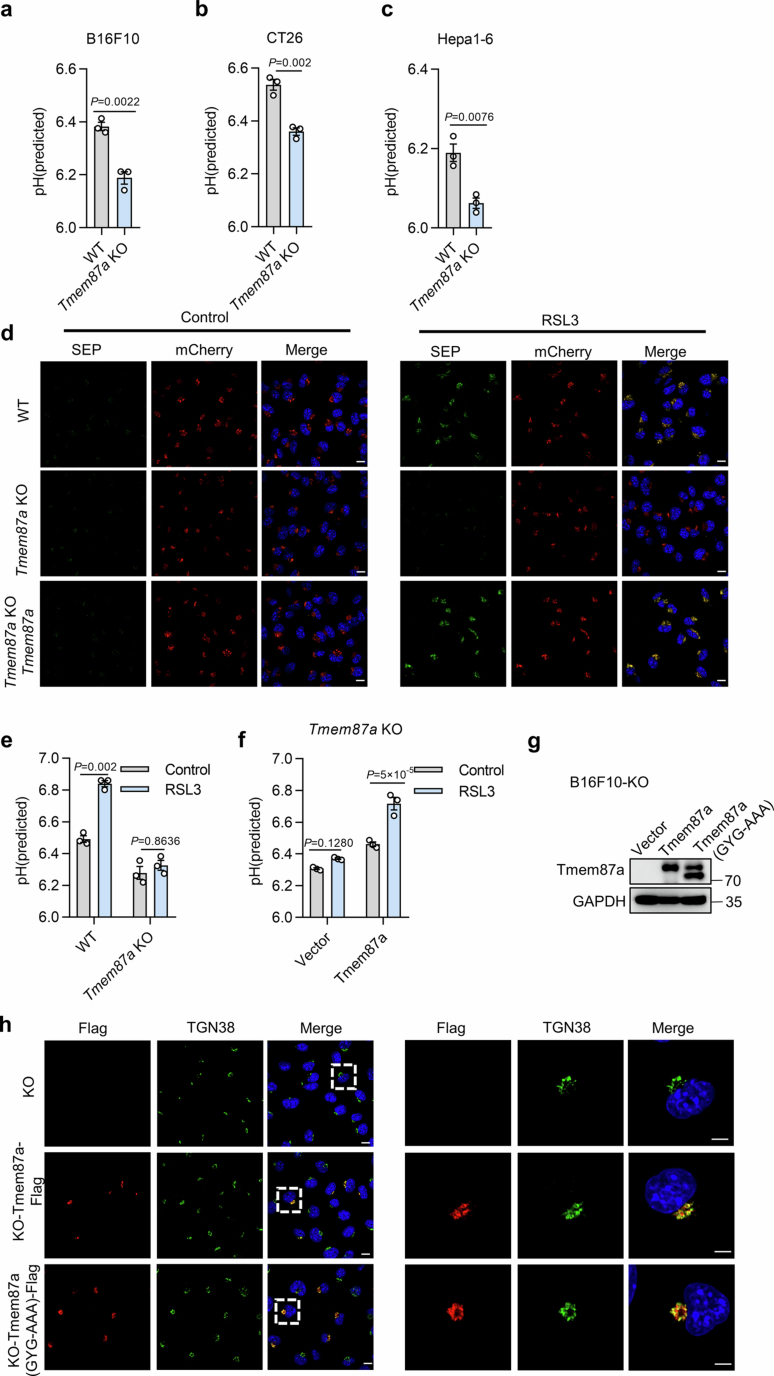
TMEM87A deficiency reverses the elevation of Golgi pH in the early stage of ferroptosis. **a- c**, Quantification of the Golgi pH in WT and *Tmem87a* KO B16F10 (**a**), CT26 (**b**) and Hepa1-6 cells (**c**). n = 3 biological replicates. **d**, Representative images of B4galt1-mCherry-SEP expressed in WT, empty vector or Tmem87a-Flag re-expressing *Tmem87a* KO B16F10 cells upon RSL3 treatment for 2 h. Scale bar, 10 μm. **e, f**, Quantification of the Golgi pH in WT and *Tmem87a* KO (**e**) or empty vector and Tmem87a-Flag re-expressing *Tmem87a* KO CT26 cells (**f**) treated with 2 μM RSL3 for 2 h. n = 3 biological replicates. **g**, Immunoblot of Tmem87a in an empty vector, Tmem87a or Tmem87a GYG-AAA mutant re-expressing *Tmem87a* KO B16F10 cells. Gapdh serves as loading control. **h**, Representative images of ectopicly expressed Tmem87a-Flag or Tmem87a (GYG-AAA)-Flag and TGN38 immunofluorescence in *Tmem87a* KO B16F10 cells. Scale bar, 10 μm (left) or 5 μm (right). All data were shown as mean ± SEM. *P* values were indicated. Statistical analyses were conducted by two-tailed t-test in (**a**- **c**) and two-way ANOVA in (**e**, **f**). Source data

Next, we studied whether Tmem87a regulated ferroptosis by modulating the Golgi pH. By expressing the Golgi pH sensor B4galt1–mCherry–SEP into WT and *Tmem87a*-KO B16F10 cells, we observed the decreased resting Golgi pH in KO cells (Extended Data Fig. 4a–c). Upon RSL3 treatment, the Golgi pH of KO cells failed to increase compared to WT cells (Fig. 2n and Extended Data Fig. 4d). Reexpression of Tmem87a–Flag into *Tmem87a*-KO cells could restore the upregulation of Golgi pH in response to RLS3 (Fig. 2o). Similar results were observed in *Tmem87a*-KO CT26 cells (Extended Data Fig. 4e,f). We then asked whether the ferroptosis sensitization caused by Tmem87a deficiency could be abrogated by elevating the Golgi pH. *Tmem87a*-KO cells reexpressing the vector control were more sensitive to RSL3 compared to those reexpressing Tmem87a–Flag (Fig. 2p) and NH_4_Cl treatment could eliminate the differences between the two cells (Fig. 2p). TMEM87A has been identified as an ion channel and the GYG motif (318–320 amino acid) is required for maintaining its activity^21^. We constructed a Tmem87a mutant with GYG substitution to AAA (GYG-AAA) and reintroduced it into WT into *Tmem87a*-KO B16F10 cells (Extended Data Fig. 4g). Reintroduction of WT Tmem87a but not the GYG-AAA mutant could elevate the Golgi pH and subsequently suppress RSL3-induced ferroptosis (Fig. 2q,r), although both cases exhibited identical Golgi localization (Extended Data Fig. 4h). Overall, these results indicate that Golgi-localized TMEM87A with channel activity mediates ferroptosis resistance through buffering the Golgi pH.

### TMEM87A protects tissues from ferroptosis in vivo

**Extended Data Fig. 5:**
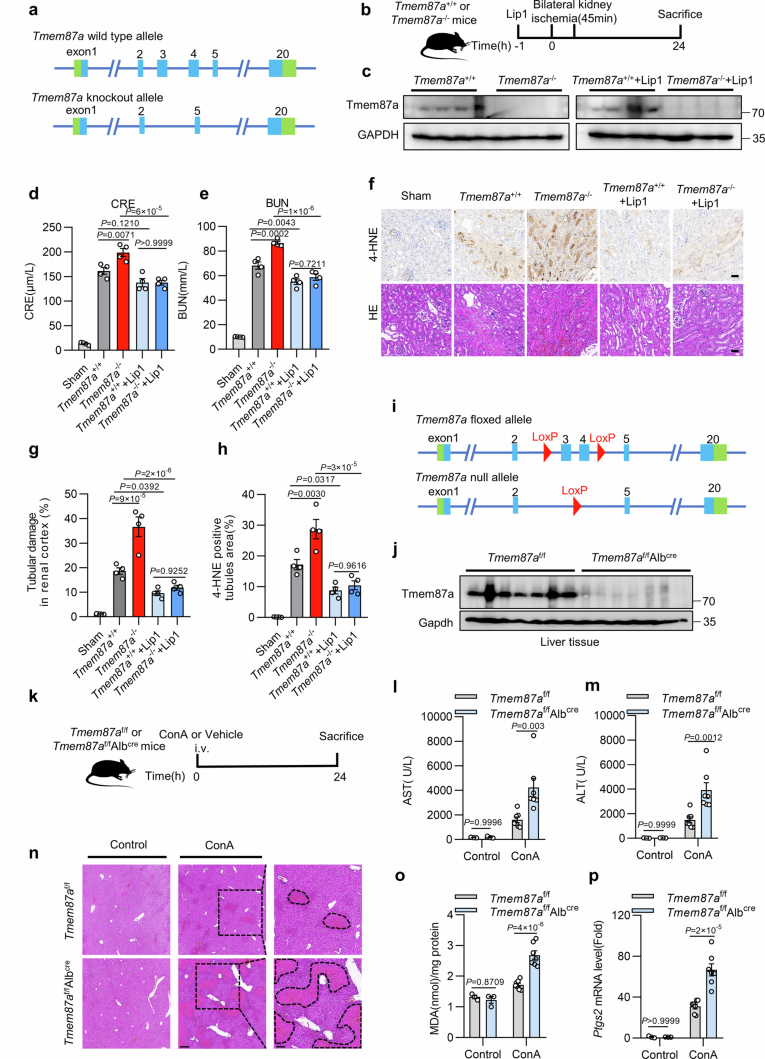
TMEM87A protects kidney and liver tissues from ferroptosis in vivo. **a**, Strategy to generate *Tmem87a* knockout (*Tmem87a*^−/−^) mice. **b- h**, Schematic of the establishment of kidney ischaemia reperfusion injury (IRI) in *Tmem87a*^+/+^ or *Tmem87a*^−/−^ mice pre-treated with liproxstatin-1 (Lip1) (**b**). Immunoblot analysis of Tmem87a expression in kidney tissues from these mice were performed (**c**). Contents of creatinine (CRE) (**d**) and blood urea nitrogen (BUN) (**e**) in orbital blood of these mice were quantified. n = 4 mice per group. IHC staining of 4-HNE (**f**, up) and H&E staining for histopathological examination (**f**, down) in kidney sections were performed. The percentages of tubular damage in renal cortex (**g**) and 4-HNE positive tubules area (**h**) in (**f**) were quantified. n = 4 mice per group. **i**, Strategy to generate *Tmem87a*^f/f^ Alb^cre^ mice. **j**, Immunoblot analysis of Tmem87a expression in livers tissues from *Tmem87a*^f/f^ and *Tmem87a*^f/f^Alb^cre^ mice. **k- p**, Schematic of the establishment of ConA induced liver damage in *Tmem87a*^f/f^ or *Tmem87a*^f/f^ Alb^cre^ mice (**k**). Contents of aspartate aminotransferase (AST) (**l**) or alanine aminotransferase (ALT) (**m**) in the orbital blood of mice were quantified. H&E staining of liver sections of mice for histopathological examination were performed (**n**). MDA content (**o**) and mRNA expression of Ptgs2 (**p**) in liver tissues of mice were quantified. n = 3 (Control) or 7 (ConA) mice per group. All data were shown as mean ± SEM. *P* values were indicated. Statistical analyses were conducted by one-way ANOVA in (**d**, **e**, **g** and **h**) and two-way ANOVA in (**l**, **m**, **o** and **p**). Source data

Our cellular findings prompted further investigation into the role of TMEM87A in ferroptosis-related pathological conditions in vivo. Renal ischemia–reperfusion injury (IRI) is a classical pathological model that is contributed by cellular ferroptosis^27^. We generated *Tmem87a*-KO (*Tmem87a*^−/−^) mice and induced renal IRI (Extended Data Fig. 5a–c). Liproxstatin 1 treatment was included to confirm the occurrence of ferroptosis (Extended Data Fig. 5b,c). Consistent with prior reports^28^, renal IRI resulted in a significant elevation of serum creatinine (CRE) and blood urea nitrogen (BUN) compared to sham controls. Furthermore, these levels were further increased in *Tmem87a*^−/−^ mice compared to WT (*Tmem87a*^+/+^) mice (Extended Data Fig. 5d,e). IRI also caused renal tubular damage and increased 4-hydroxynonenal (4-HNE) staining, which were further aggravated in the kidneys from *Tmem87a*^−/−^ mice (Extended Data Fig. 5f–h). Notably, liproxstatin 1 administration reduced all pathological parameters and eliminated differences between KO and WT groups (Extended Data Fig. 5d–h). Therefore, we conclude that Tmem87a protects the kidney from IRI-induced renal cell ferroptosis.

Concanavalin A (ConA)-induced acute liver injury is another model that involves hepatic ferroptosis^29^. We then generated hepatocyte-specific *Tmem87a*-KO mice (*Tmem87a*^f/f^Alb^cre^) and treated them and control *Tmem87a*^f/f^ mice with a sublethal dose of ConA (Extended Data Fig. 5i–k). The liver damage of mice was revealed by the increased serum levels of aspartate aminotransferase (AST) and alanine aminotransferase (ALT) and their levels were even higher in the serum from *Tmem87a*^f/f^Alb^cre^ mice comparing with the one from *Tmem87a*^f/f^ mice (Extended Data Fig. 5l,m). Consistent with this, histological analyses revealed more extensive necrotic area in liver tissue from *Tmem87a*^f/f^Alb^cre^ mice (Extended Data Fig. 5n). In addition, ConA treatment in *Tmem87a*^f/f^Alb^cre^ mice resulted in elevated malondialdehyde (MDA) content (Extended Data Fig. 5o) and expression of a ferroptosis marker Ptgs2 (Extended Data Fig. 5p) compared to *Tmem87a*^f/f^ mice. Together, these data suggest that hepatic Tmem87a deficiency aggravates ferroptosis-related liver damage.

**Extended Data Fig. 6:**
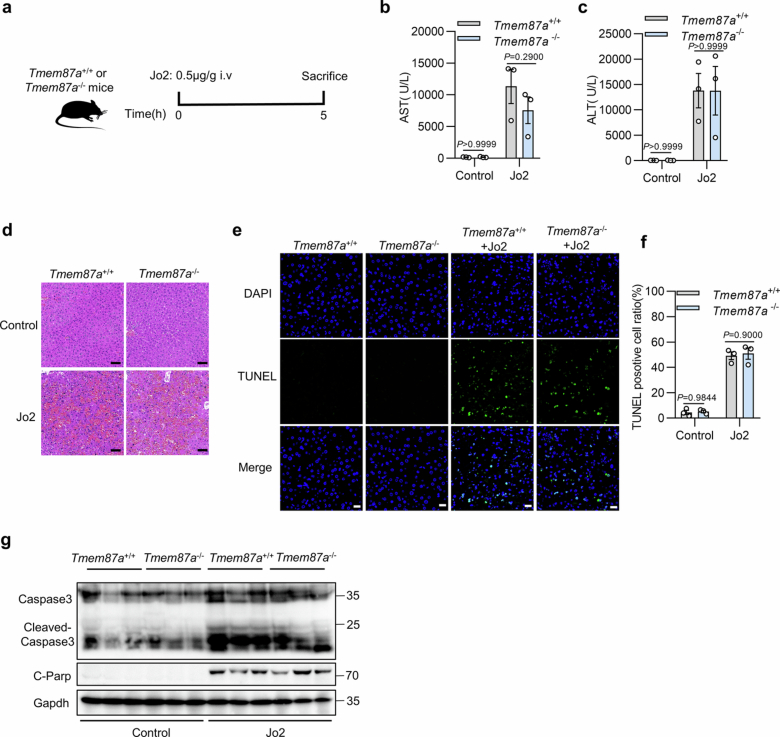
TMEM87A deficiency does not impact Jo2-induced liver apoptosis in vivo. **a- g**, Schematic of the establishment of Jo2-induced liver apoptosis model in *Tmem87a*^+/+^ or *Tmem87a*^−/−^ mice (**a**). Contents of AST (**b**) or ALT (**c**) level in the orbital blood of these mice were quantified. n = 3 mice per group. H&E staining for histopathological examination in liver tissues from these mice were performed (**d**). Scale bar: 50 μm. TUNEL staining of liver tissues from these mice were performed (**e**) and TUNEL positive cell ratios were quantified (**f**). Scale bar, 20 μm. n = 3 mice per group. Immunoblot of Caspase3 and C-PARP in liver tissues from these mice were performed (**g**). All data were shown as mean ± SEM. *P* values were indicated. Statistical analyses were conducted by two-way ANOVA (**b**, **c** and **f**). Source data

We also examined whether *Tmem87a* KO affected apoptosis-mediated liver damage through the administration of an anti-Fas monoclonal antibody (Jo2)^30^ (Extended Data Fig. 6a). Jo2 treatment caused significant increases in AST and ALT levels (Extended Data Fig. 6b,c). However, no differences were detected between *Tmem87a*^+/+^ and *Tmem87a*^−/−^ mice (Extended Data Fig. 6b,c). Furthermore, we examined the pathology, cell death and cleavage of caspase 3 and Parp in Jo2-treated liver tissues and no differences were observed between the two groups (Extended Data Fig. 6d–g). Therefore, Tmem87a deficiency may not affect apoptotic cell death induced by Fas activation.

### TMEM87A ablation promotes ferroptosis by causing FSP1 condensation

**Fig. 3:**
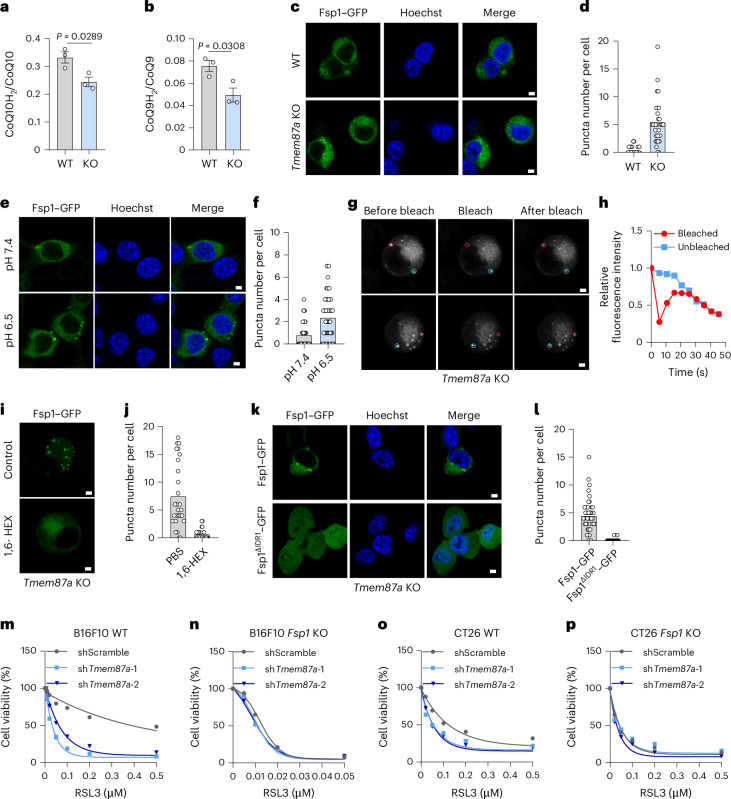
TMEM87A deficiency sensitizes tumor cells to ferroptosis through promoting FSP1 condensation. **a**,**b**, The ratio of CoQ10H_2_ to CoQ10 content (**a**) and CoQ9H_2_ to CoQ9 content (**b**) in WT and *Tmem87a*-KO CT26 cells (*n* = 3 biological replicates). **c**,**d**, Representative images of the distribution of Fsp1–GFP in WT and *Tmem87a*-KO CT26 cells (**c**) and the number of Fsp1–GFP puncta per cell (**d**). Scale bar, 10 μm (n = 48 (WT) or 30 (*Tmem87*-KO) cells). The data shown are representative results from three independent experiments with similar results. **e**,**f**, Representative images of the distribution of Fsp1–GFP in CT26 cells incubated with pH 7.4 or pH 6.5 culture medium (**e**) and quantification of Fsp1–GFP puncta number (**f**). Scale bar, 10 μm (*n* = 55 cells (pH 7.4 and pH 6.5)). The data shown are representative results from three independent experiments with similar results. **g**,**h**, Representative images of FRAP assay in *Tmem87a*-KO CT26 cells stably expressing Fsp1–GFP. Red and blue circles represent the bleached and control, respectively (**g**). Scale bar, 10 μm. Quantification of relative fluorescence intensity of FRAP (**h**) (*n* = 3 technical replicates). The data shown are representative results from three independent experiments with similar results. **i**,**j**, Representative images of the Fsp1 distribution before and after treatment with 10% 1,6-HEX for 5 min in *Tmem87a*-KO CT26 cells stably expressing Fsp1–GFP (**i**) and quantification of Fsp1–GFP puncta number (**j**). Scale bar, 10 μm (*n* = 27 (PBS) or 24 (1,6-HEX) cells). The data shown are representative results from three independent experiments with similar results. **k**,**l**, Representative images of the distribution of Fsp1–GFP or Fsp1^ΔIDR1^–GFP in *Tmem87a*-KO CT26 cells (**k**) and quantification of GFP puncta number per cell (**l**). Scale bar, 10 μm (*n* = 44 (Fsp1–GFP) or 73 (Fsp1^ΔIDR1^–GFP) cells). The data shown are representative results from three independent experiments with similar results. **m**–**p**, Relative cell viability of WT and *Fsp1*-KO B16F10 cells (**m**,**n**) or CT26 cells (**o**,**p**) expressing shScramble and sh*Tmem87a* treated with RSL3 (*n* = 3 technical replicates in the conditions of B16F10 *Fsp1*-KO shScramble RSL3 (0.01, 0.02 and 0.05 µM) and CT26 *Fsp1*-KO shScramble RSL3 (0.02 and 0.05 µM); *n* = 4 technical replicates for all other conditions). Data shown are the mean (**d**,**f**,**h**,**j**,**l**–**p**) or mean ± s.e.m. (**a**,**b**). *P* values are indicated. Statistical analyses were conducted using a two-tailed *t*-test (**a**,**b**). Source data

**Extended Data Fig. 7:**
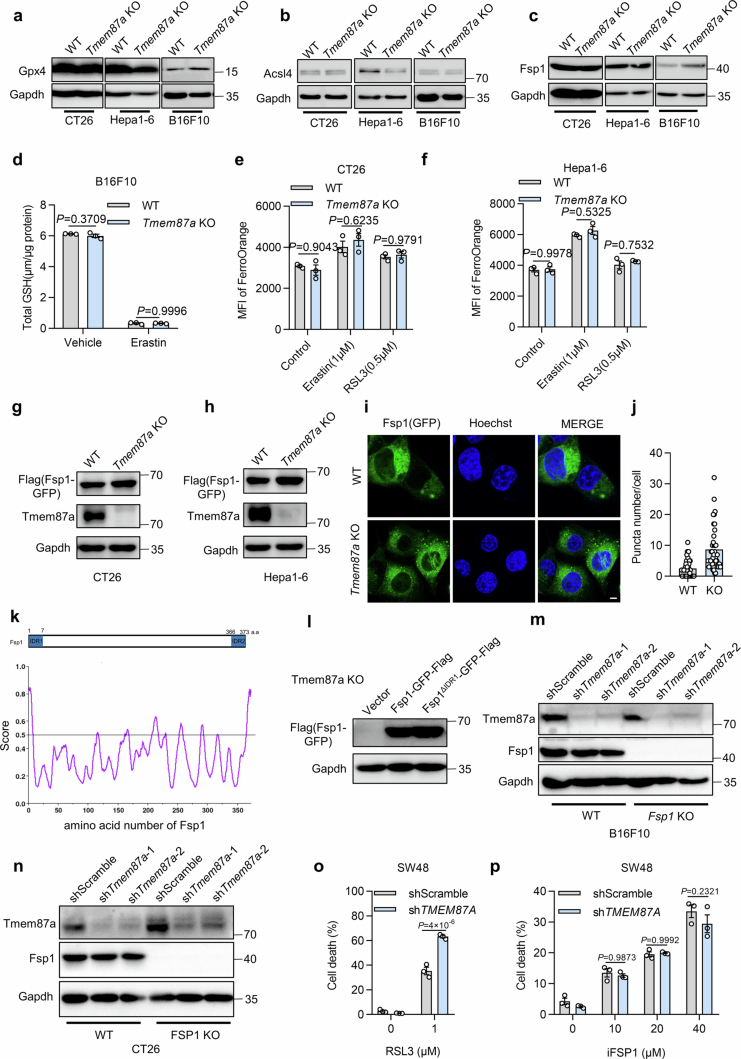
TMEM87A modulates tumor cell ferroptosis through regulating the condensation of FSP1. **a- c**, Immunoblot of Gpx4 (**a**), Acsl4 (**b**), Fsp1 (**c**) in WT and *Tmem87a* KO CT26, Hepa1-6 and B16F10 cells. Gapdh serves as a loading control. **d**, Quantification of total GSH in WT and *Tmem87a* KO B16F10 cells treated with Erastin (1 μM for 8 h). n = 3 biological replicates. **e, f**, Intracellular iron ions were bound by iron sensing dye FerroOrange and detected in WT and *Tmem87a* KO CT26 (**e**) or Hepa1-6 cells (**f**) treated with Erastin (1 μM) or RSL3 (0.5 μM). n = 3 biological replicates. **g, h**, Immunoblot of Tmem87a and Flag-tagged Fsp1-GFP in Flag-tagged Fsp1-GFP overexpressing WT and *Tmem87a* KO CT26 (**g**) or Hepa1-6 cells (**h**). Gapdh serves as loading control. **i, j**, Representative images of the distribution of Fsp1-GFP in WT and *Tmem87a* KO Hepa1-6 cells (**i**). Scale bar, 10 μm. The Fsp1-GFP puncta number was quantified (**j**). n = 36 cells (WT) or 41 cells (KO). The data were shown as representative results from three independent experiments with similar results. **k**, Predictions of intrinsically disordered regions (IDR) of mouse Fsp1 by PONDR algorithms. Schematic representation of mouse FSP1 protein structure is shown above. There are two IDRs (1-7, 366-373 amino acid) in mouse Fsp1. **l**, Immunoblot of Flag-tagged Fsp1 in empty vector, Fsp1-GFP-Flag or Fsp1^ΔIDR1^-GFP-Flag overexpressing *Tmem87a* KO CT26 cells. Gapdh serves as loading control. **m, n**, Immunoblot of Tmem87a and Fsp1 in WT and *Fsp1* KO B16F10 (**m**) or CT26 cells (**n**) expressing shScramble or Tmem87a-targeting shRNA. Gapdh serves as loading control. **o, p**, Cell death of SW48 cells expressing shScramble or TMEM87A-targeting shRNA treated with RSL3 (**o**) or iFSP1 (**p**). n = 3 biological replicates. Data were shown as mean (**j**) or mean ± SEM (**d** - **f**, **o** - **p**). *P* values were indicated. Statistical analyses were conducted by two-way ANOVA in (**d**-**f**, **o**, **p**). Source data

To elucidate how TMEM87A regulates ferroptosis resistance, we first analyzed the relative abundances of key proteins and metabolites that are known to regulate ferroptosis in WT and *Tmem87a*-KO cells. The protein levels of Gpx4, Acsl4 and Fsp1 were all similar between WT and *Tmem87a*-KO cells (Extended Data Fig. 7a–c). The intracellular contents of GSH and labile iron(II) ions (Fe^2+^) were not affected by Tmem87a deficiency (Extended Data Fig. 7d–f). Surprisingly, a pronounced reduction of intracellular CoQH_2_, as revealed by the lower ratios of CoQ10H_2_/CoQ10 and CoQ9H_2_/CoQ9, was detected in *Tmem87a*-KO cells compared to WT cells (Fig. 3a,b). FSP1 is the only known oxidoreductase that reduces ubiquinone to its hydroquinone form^31,32^. Considering that Tmem87a deficiency has no effect on Fsp1 expression, we speculated that the enzymatic activity of Fsp1 might be disturbed. Given the importance of FSP1 subcellular localization in its antiferroptotic activity^33^, we examined the distribution of mouse Fsp1–GFP in WT and *Tmem87a*-KO cells. Although the amounts of Fsp1–GFP protein were similar between WT and *Tmem87a*-KO cells (Extended Data Fig. 7g,h), more puncta of Fsp1–GFP were observed in *Tmem87a*-KO cells (Fig. 3c,d and Extended Data Fig. 7i,j). Furthermore, incubation of cells with acidic culture medium resulted in the increased formation of Fsp1–GFP puncta (Fig. 3e,f). As human FSP1 forms condensates through phase separation^33^ and mouse Fsp1 also contains two predicted intrinsically disordered regions (IDRs) (Extended Data Fig. 7k), we speculated that the above Fsp1–GFP puncta might be phase-separated condensates. Upon photobleaching, the fluorescence of Fsp1–GFP condensates recovered within several seconds (Fig. 3g,h). Treatment with 1,6-hexanediol (1,6-HEX), a chemical known to generally disrupt liquid-like condensates^34^, resulted in an obvious reduction in Fsp1–GFP condensates (Fig. 3i,j). Moreover, an IDR-truncated Fsp1^ΔIDR1^ failed to form puncta in *Tmem87a*-KO cells (Fig. 3k,l and Extended Data Fig. 7l). These data suggest that Tmem87a deficiency causes the condensation of mouse Fsp1.

To further examine whether Tmem87a regulates ferroptosis through Fsp1, we knocked out *Fsp1* in B16F10 cells followed by *Tmem87a* knockdown (Extended Data Fig. 7m). While *Tmem87a* knockdown sensitized WT cells to RSL3 (Fig. 3m), this effect was largely abolished in *Fsp1*-KO cells (Fig. 3n). Consistent results were obtained in CT26 cells (Fig. 3o,p and Extended Data Fig. 7n). Given that currently available FSP1 inhibitors are selective for human FSP1, we also performed the experiments on human SW48 tumor cells. *TMEM87A* knockdown could increase RSL3-induced ferroptosis but had no effect on cell death induced by a well-documented human FSP1 inhibitor (iFSP1) (Extended Data Fig. 7o,p). These findings indicate that FSP1 is required for TMEM87A inhibition-mediated ferroptosis sensitization.

### TMEM87A loss impairs tumor growth in vivo

**Fig. 4:**
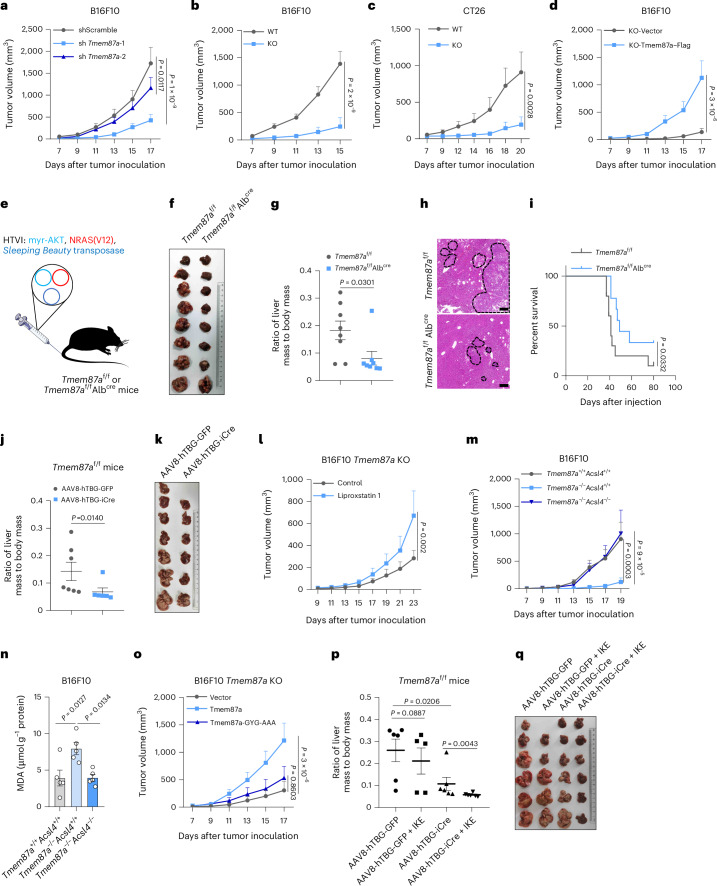
TMEM87A deficiency impairs tumor growth in vivo*.* **a**, Tumor volumes of shScramble or sh*Tmem87a*-expressing B16F10 cells inoculated in C57BL/6 mice (*n* = 8 mice per group). **b**,**c**, Tumor volumes of WT and *Tmem87a*-KO B16F10 (**b**) and CT26 (**c**) cells inoculated in C57BL/6 or BALB/c mice (**b**, *n* = 8 (WT) or 7 (*Tmem87a*-KO) mice per group; **c**, *n* = 8 (WT) or 6 (*Tmem87a*-KO) mice per group). **d**, Tumor volumes of *Tmem87a*-KO B16F10 cells reexpressing Tmem87a–Flag or an empty vector inoculated in C57BL/6 mice (*n* = 6 (KO + empty vector) or 8 (KO + Tmem87a–Flag) mice per group). **e**–**i**, Effect of Tmem87a deficiency on the progression of orthotopic HCC. Schematic of the establishment of AKT–NRAS hydrodynamic tail-vein injection-induced orthotopic HCC model in *Tmem87a*^f/f^ or *Tmem87a*^f/f^Alb^cre^ mice (**e**). Representative images of intrahepatic tumor burden after 5 weeks (**f**). The ratio of tumor-bearing liver mass to body mass in mice that were weighed at the end point (*n* = 8 per group) (**g**). Representative H&E staining for histopathological examination (**h**). Kaplan–Meier survival curves of *Tmem87a*^f/f^ (*n* = 10) and *Tmem87a*^f/f^Alb^cre^ (*n* = 9) mice with orthotopic HCC (**i**). **j**,**k**, The ratio of tumor-bearing livers to body mass of *Tmem87a*^f/f^ mice with AAV8-hTBG-GFP (n = 7) or AAV8-hTBG-iCre (n = 6) injection (**j**). Representative images of intrahepatic tumor burden (**k**). **l**, Tumor volumes of *Tmem87a*-KO B16F10 cells inoculated in C57BL/6 mice treated with liproxstatin 1 (*n* = 7) or control (*n* = 6). **m**, Tumor volumes of *Tmem87a*^+/+^*Acsl4*^+/+^ (*n* = 7), *Tmem87a*^−/−^*Acsl4*^+/+^ (*n* = 7) and *Tmem87a*^−/−^*Acsl4*^−/−^ (*n* = 6) B16F10 cells inoculated in C57BL/6 mice. **n**, MDA content in *Tmem87a*^+/+^*Acsl4*^+/+^, *Tmem87a*^−/−^*Acsl4*^+/+^ and *Tmem87a*^−/−^*Acsl4*^−/−^ B16F10 tumor tissues isolated from C57BL/6 mice (*n* = 5 mice per group). **o**, Tumor volumes of *Tmem87a*-KO B16F10 cells reexpressing empty vector (*n* = 10), Tmem87a (*n* = 9) or Tmem87a-GYG-AAA mutant (*n* = 7) inoculated in C57BL/6 mice. **p**,**q**, The ratio of tumor-bearing liver mass to body mass (**p**) and representative images of intrahepatic tumor burden (**q**) isolated from *Tmem87a*^f/f^ mice with AAV8-hTBG-GFP or AAV8-hTBG-iCre injection plus IKE treatment (*n* = 5 mice in the AAV8-hTBG-GFP + IKE group; *n* = 6 mice in all other groups). All data shown are the mean ± s.e.m. *P* values are indicated. Statistical analyses were conducted using a two-way ANOVA (**a**–**d**,**l**,**m**,**o**), two-tailed *t*-test (**g**,**j**), log-rank test (**i**) or one-way ANOVA (**n**,**p**). Source data

**Extended Data Fig. 8:**
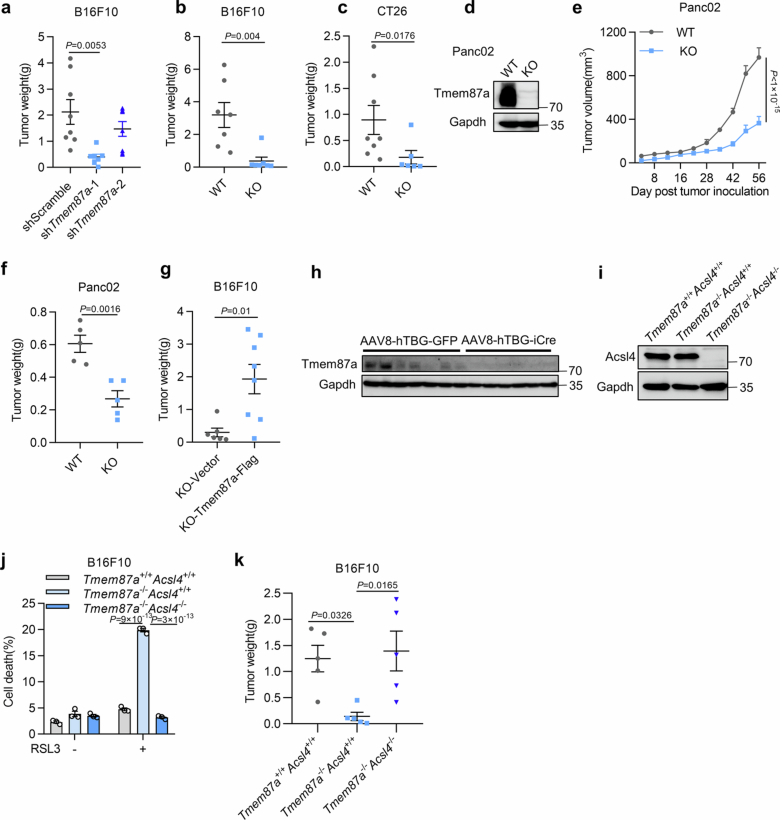
TMEM87A loss inhibits tumor progression. **a**, The weights of B16F10 tumors expressing shScramble (n = 8 mice), shTMEM87a-1 (n = 7 mice) or shTMEM87a-2 (n = 8 mice) isolated from C57BL/6 mice. **b**, The weights of WT (n = 7 mice) and *Tmem87a* KO (n = 7 mice) B16F10 tumors. **c**, The weights of WT (n = 8 mice) and *Tmem87a* KO (n = 6 mice) CT26 tumors. **d**, Immunoblot of Tmem87a in WT and *Tmem87a* KO Panc02 cells. Gapdh serves as loading control. **e**, Tumor volumes of WT and *Tmem87a* KO Panc02 cells inoculated in C57BL/6 mice. n = 5 mice per group. **f**, The weights of WT and *Tmem87a* KO Panc02 tumors isolated from C57BL/6 mice. n = 5 mice per group. **g**, The weights of an empty vector (n = 6 mice) or Tmem87a-Flag (n = 8 mice) re-expressing *Tmem87a* KO B16F10 tumors isolated from C57BL/6 mice. **h**, Immunoblot of liver tissues isolated from *Tmem87a*^f/f^ mice with AAV8- hTBG-GFP or AAV8-hTBG-iCre injection. Gapdh serves as loading control. **i**, Immunoblot of Ascl4 in *Tmem87a*^+/+^*Acsl4*^+/+^, *Tmem87a*^−/−^*Acsl4*^+/+^, *Tmem87a*^−/−^*Acsl4*^−/−^ B16F10 cells. Gapdh serves as loading control. **j**, Cell death of *Tmem87a*^+/+^*Acsl4*^+/+^, *Tmem87a*^−/−^*Acsl4*^+/+^, *Tmem87a*^−/−^*Acsl4*^−/−^ B16F10 cells treated with RSL3. n = 3 biological replicates. **k**, The weights of *Tmem87a*^+/+^*Acsl4*^+/+^, *Tmem87a*^−/−^*Acsl4*^+/+^, *Tmem87a*^−/−^*Acsl4*^−/−^ B16F10 tumors isolated C57BL/6 mice. n = 5 mice per group. All data were shown as mean ± SEM. *P* values were indicated. Statistical analyses were conducted by one-way ANOVA in (**a** and **k**), two-tailed t-test in (**b**, **c**, **f** and **g**) and two-way ANOVA in (**e** and **j**). Source data

To further investigate the function of TMEM87A in tumor progression, we inoculated *Tmem87a*-knockdown or *Tmem87a*-KO B16F10 cells into immunocompetent C57BL/6 mice. *Tmem87a* knockdown or KO significantly suppressed tumor growth, as shown by both tumor volume (Fig. 4a,b) and weight (Extended Data Fig. 8a,b) compared to control tumors. The tumor-suppressive effect of Tmem87a ablation was consistently observed across multiple tumor models including CT26 and Panc02 (Fig. 4c and Extended Data Fig. 8c–f). As further confirmation, reexpression of Tmem87a–Flag in *Tmem87a*-KO B16F10 cells restored the tumor growth (Fig. 4d and Extended Data Fig. 8g). Next, we tested whether Tmem87a is required for the development of orthotopic hepatocellular carcinoma (HCC), in which three plasmids encoding myristoylated AKT, NRAS(V12) and *Sleeping Beauty* transposon were codelivered into the liver through hydrodynamic tail-vein injection (Fig. 4e). We observed fewer tumors on the liver tissues of *Tmem87a*^f/f^Alb^cre^ mice compared to *Tmem87a*^f/f^ mice (Fig. 4f–h). Accordingly, *Tmem87a*^f/f^Alb^cre^ mice with HCC showed longer survival (Fig. 4i). Moreover, we tested whether inducible KO of *Tmem87a* after HCC establishment could still delay cancer progression. AAV8-hTBG-iCre administration achieved liver-specific inducible KO of *Tmem87a* in *Tmem87a*^f/f^ mice (Extended Data Fig. 8h) and caused a significant reduction in tumor burden, as evidenced by lower ratios of liver mass to body mass (Fig. 4j,k). Taken together, these results indicate that Tmem87a ablation inhibits the progression across multiple tumor types in vivo.

Next, to examine whether ferroptosis susceptibility confers to Tmem87a loss-mediated tumor suppression, we treated mice bearing *Tmem87a*-KO B16F10 tumor with liproxstatin 1 and found that tumor growth was promoted (Fig. 4l). To specifically inhibit ferroptosis of tumor cells, we knocked out long-chain acyl-CoA synthetase 4 (*Acsl4*), which is required for ferroptosis initiation^35^, in *Tmem87a*-KO B16F10 cells (Extended Data Fig. 8i). Acsl4 deficiency could restore the cellular resistance to RSL3 (Extended Data Fig. 8j). In vivo, Acsl4 deficiency fully neutralized the tumor-suppressive effect mediated by *Tmem87a* KO (Fig. 4m and Extended Data Fig. 8k) and reversed the induction of tumoral MDA content (Fig. 4n). More importantly, as the GYG motif of Tmem87a is required for its regulation of ferroptosis, we found that the reexpression of WT but not the GYG-AAA mutant could restore the growth of *Tmem87a*-KO tumors in vivo (Fig. 4o). These data indicate that Tmem87a deficiency impairs tumor growth by promoting tumoral ferroptosis.

Lastly, we wondered whether Tmem87a deficiency could augment ferroptosis-targeting therapy. Orthotopic HCC tumors with inducible KO of hepatic *Tmem87a* were established in *Tmem87a*^f/f^ mice followed by IKE treatment. IKE significantly inhibited the growth of HCC tumor with *Tmem87a* KO but had no effect on Tmem87a-expressing control tumors (Fig. 4p,q), suggesting that Tmem87a ablation synergizes with ferroptosis inducer to exert antitumor activity.

### TMEM87A ablation boosts antitumor CD8^+^ T cell response

**Fig. 5:**
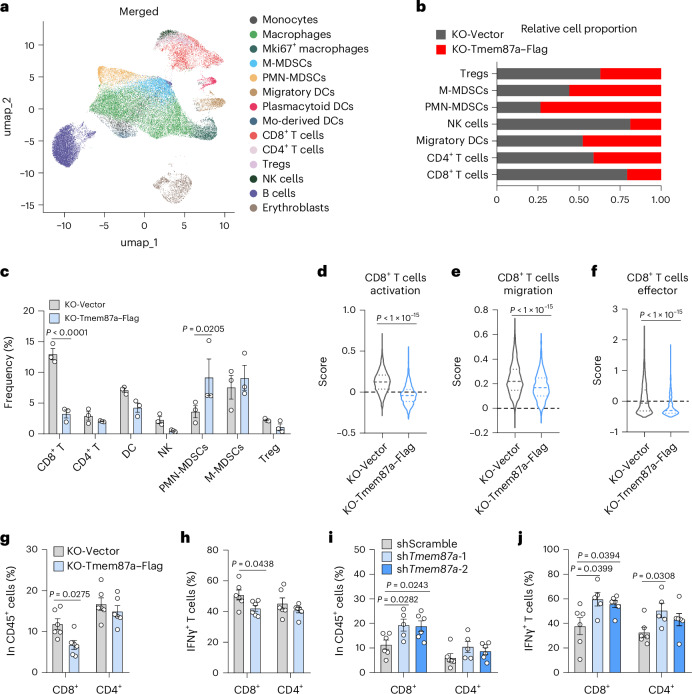
TMEM87A deficiency enhances antitumor T cell response. **a**–**f**, scRNA-seq analysis of CD45^+^ cells from *Tmem87a*-KO B16F10 tumors reexpressing empty vector or Tmem87a–Flag in C57BL/6 mice. The CD45^+^ tumor-infiltrating leukocytes were sorted by flow cytometry for 10x genomic scRNA-seq (*n* = 3 mice per group). Merged UMAP plot (**a**). Relative cell proportions (**b**) and frequency (**c**) of immune subpopulations in *Tmem87a*-KO + empty vector or *Tmem87a*-KO + Tmem87a–Flag tumors. Transcripts from CD8^+^ T cells in tumors were assessed for signature enrichment of CD8^+^ T cell activation (**d**), migration (**e**) and effector function (**f**). **g**,**h**, Immune cells infiltration in *Tmem87a*-KO B16F10 tumors reexpressing empty vector or Tmem87a–Flag detected by flow cytometry (*n* = 6 independent tumors per group). Percentages of CD8^+^ and CD4^+^ T cell subsets in CD45^+^ population (**g**). Percentages of IFNγ^+^ cells subsets in CD8^+^ T cells and CD4^+^ T cells (**h**). **i**,**j**, Immune cell infiltration in B16F10 tumors expressing Scramble or *Tmem87a*-targeting shRNA detected by flow cytometry (*n* = 6 independent tumors per group, except for the sh*Tmem87a*-1 group, where *n* = 5 tumors). Percentages of CD8^+^ and CD4^+^ T cells subsets in CD45^+^ population (**i**). Percentages of IFNγ^+^ cells subsets in CD8^+^ T cells and CD4^+^ T cells (**j**). All data shown are the mean ± s.e.m. *P* values are indicated. Statistical analyses were conducted using a two-way ANOVA (**c**,**g**–**j**) or Mann–Whitney test (**d**–**f**). Source data

Given the proinflammatory potential of ferroptosis, we investigated whether Tmem87a loss reshapes the tumor immune microenvironment by performing single-cell RNA sequencing (scRNA-seq) on CD45^+^ cells enriched from B16F10 *Tmem87a*-KO + empty vector and *Tmem87a*-KO + Tmem87a–Flag tumors (Fig. 5a). *Tmem87a*-KO + empty vector tumors exhibited a shifted immune landscape, characterized by an expansion of antitumor CD8^+^ T cells and a reduction in immunosuppressive polymorphonuclear myeloid-derived suppressor cells (PMN-MDSCs) (Fig. 5b,c). The scores for CD8^+^ T cell activation, migration and effector function were also higher in the *Tmem87a*-KO + empty vector group (Fig. 5d–f). Flow cytometry analysis validated the increased CD8^+^ T cells and elevated IFNγ production in *Tmem87a*-KO + empty vector tumors (Fig. 5g,h), as well as in *Tmem87a*-knockdown tumors (Fig. 5i,j). These data suggest that tumoral Tmem87a ablation results in a stronger antitumor CD8^+^ T cell response.

**Extended Data Fig. 9:**
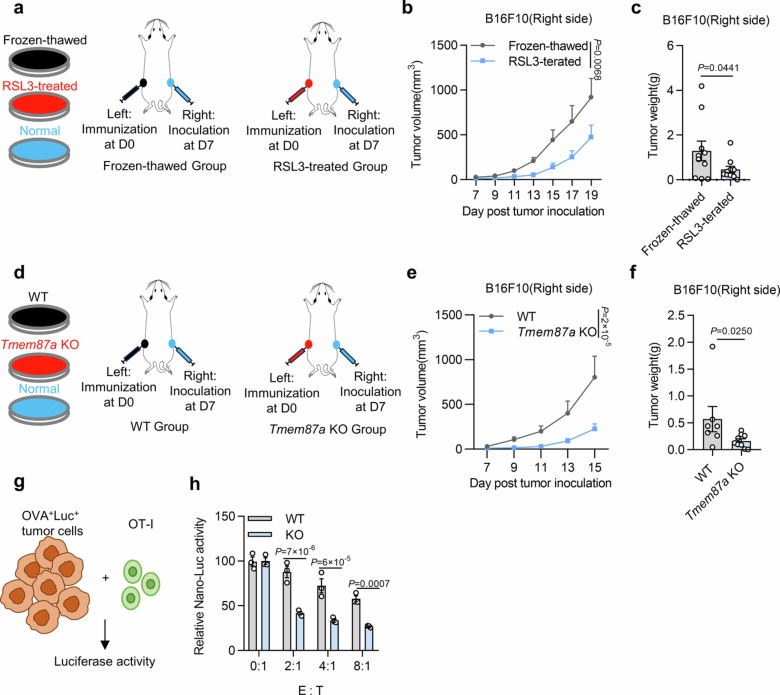
Ferroptotic TMEM87A deficient tumor cells exhibit enhanced immunogenicity. **a- c**, Experimental design for evaluating the immunogenicity of RSL3-induced ferroptotic B16F10 cells compared to frozen-thawed cells in C57BL/6 mice (**a**). The growth of B16F10 tumor inoculated on right flank was monitored (**b**). The weights of these isolated tumors were shown (**c**). n = 10 mice per group. **d- f**, Experimental design for evaluating the immunogenicity of *Tmem87a* KO B16F10 cells compared to WT cells in C57BL/6 mice (**d**). The growth of B16F10 tumor inoculated on right flank was monitored (**e**). The weights of these isolated tumors were shown (**f**). n = 7 (WT) or 9 (KO) mice per group. **g**, Schematic illustration of OVA^+^ Luc^+^ tumor cells co-cultured with OT-l cells. **h**, Relative Nano-luciferase activity of OVA^+^ Luc^+^ tumor cells that were co-cultured with OT-l cells for 24 h at the indicated effector-to-target ratio. All Data were shown as mean ± SEM. *P* values are determined by two-way ANOVA in (**b**, **e** and **h**) and two-tailed t-test in (**c** and **f**). Source data

We then tested the immunogenicity of ferroptotic Tmem87a-deficient tumor cells. We immunized mice with freeze–thaw-treated or RSL3-induced ferroptotic B16F10 cells and rechallenged them with live tumor cells (Extended Data Fig. 9a). By monitoring the rechallenged tumor growth, we found a significant reduction in mice immunized with ferroptotic cells compared to freeze–thaw-treated group (Extended Data Fig. 9b,c). Next, we compared the immunogenicity of WT and *Tmem87a*-KO B16F10 tumors using a similar experimental design (Extended Data Fig. 9d). The results showed that immunization with *Tmem87a*-KO cells was more efficient at suppressing the growth of rechallenged tumor (Extended Data Fig. 9e,f). Overall, these results suggest that ferroptosis induction, through either RSL3 treatment or Tmem87a ablation, enhances tumoral immunogenicity, which may contribute to an improved antitumor immune response.

CD8^+^ T cells can promote tumor ferroptosis, which underlies its antitumor activity^15,16^. We then tested whether Tmem87a-deficient tumor cells would become more sensitive to CD8^+^ T cell-mediated killing. We established WT and *Tmem87a*-KO B16F10 cells coexpressing ovalbumin (OVA) and luciferase (Luc). After coculturing them with OVA-specific CD8^+^ T (OT-I) cells, tumor cell viability was evaluated by quantifying of Luc activity (Extended Data Fig. 9g). *Tmem87a*-KO tumor cells were more vulnerable to OT-I-mediated killing compared to WT cells (Extended Data Fig. 9h), suggesting that Tmem87a deficiency sensitizes tumor cells to CD8^+^ T cells.

### TMEM87A deficiency sensitizes tumor to PD1 blockade therapy

**Fig. 6:**
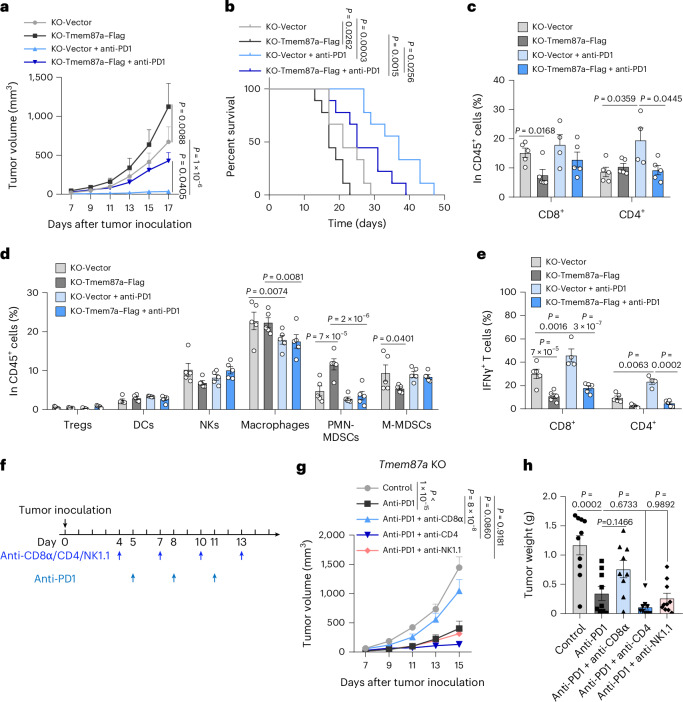
TMEM87A deficiency sensitizes tumor to immunotherapy. **a**,**b**, Tumor volumes of *Tmem87a*-KO + empty vector or *Tmem87a*-KO + Tmem87a–Flag B16F10 cells inoculated in C57BL/6 mice treated with anti-PD1 antibody (**a**) (*n* = 9 mice per group, except for the *Tmem87a*-KO + empty vector anti-PD1 group, where *n* = 8 mice). The Kaplan–Meier survival curves of these tumor-bearing mice (**b**). **c**–**e**, Leukocyte infiltration in *Tmem87a*-KO + empty vector or *Tmem87a*-KO + Tmem87a–Flag B16F10 tumors treated with anti-PD1 antibody detected by flow cytometry. Percentages of CD8^+^ and CD4^+^ T cells subsets in CD45^+^ population (**c**) (*n* = 5 independent tumors per group, except for the *Tmem87a*-KO + empty vector anti-PD1 group, where *n* = 4 tumors). Percentages of different immune cells subpopulations including regulatory T cells, dendritic cells, NK cells, macrophages, PMN-MDSCs and M-MDSCs in the CD45^+^ population (**d**) (*n* = 5 independent tumors per group). Percentages of IFNγ^+^ cells subsets in CD8^+^ T cells and CD4^+^ T cells (**e**) (*n* = 5 independent tumors per group, except for the *Tmem87a*-KO + empty vector anti-PD1 group, where *n* = 4 tumors). **f**, Experimental design of *Tmem87a*-KO B16F10 cells inoculated in C57BL/6 mice treated with anti-CD8α, anti-CD4 or anti-NK1.1 antibody followed by anti-PD1 therapy. **g**,**h**, Tumor volumes of *Tmem87a*-KO B16F10 cells inoculated in C57BL/6 mice treated with anti-PD1 or anti-PD1 combined with anti-CD8α, anti-CD4 or anti-NK1.1 antibody (**g**). The weights of tumors isolated from these mice (**h**) (*n* = 10 mice in control and anti-PD1 + anti-NK1.1 groups; *n* = 9 mice in the other groups). All data shown are the mean ± s.e.m. *P* values are indicated. Statistical analyses were conducted using a two-way ANOVA (**a**,**c**–**e**,**g**), log-rank test (**b**) or one-way ANOVA (**h**). Source data

Ferroptosis-targeting therapy combined with ICB has shown synergistic antitumor activity^15,36–38^. Thus, we tested whether tumoral Tmem87a ablation would augment the efficacy of PD1 blockade therapy. We inoculated *Tmem87a*-KO B16F10 tumors reexpressing empty vector or Tmem87a–Flag in C57BL/6 mice and treated them with anti-PD1 antibody. As expected, *Tmem87a*-KO + empty vector tumors were more sensitive to the therapy, as revealed by significantly decreased tumor volume and improved animal survival (Fig. 6a,b). Consistent with this, *Tmem87a*-KO + empty vector tumors exhibited increased infiltration of CD8^+^ T cells and decreased PMN-MDSCs compared to *Tmem87a*-KO + Tmem87a–Flag tumors and PD1 blockade therapy further elevated T cell infiltration while reducing PMN-MDSCs in *Tmem87a*-KO + empty vector tumors (Fig. 6c,d). Moreover, both CD8^+^ and CD4^+^ T cells from PD1 blockade-treated *Tmem87a*-KO + empty vector tumors exhibited the highest IFNγ production (Fig. 6e). These results indicate that Tmem87a ablation augments PD1 blockade therapy.

**Extended Data Fig. 10:**
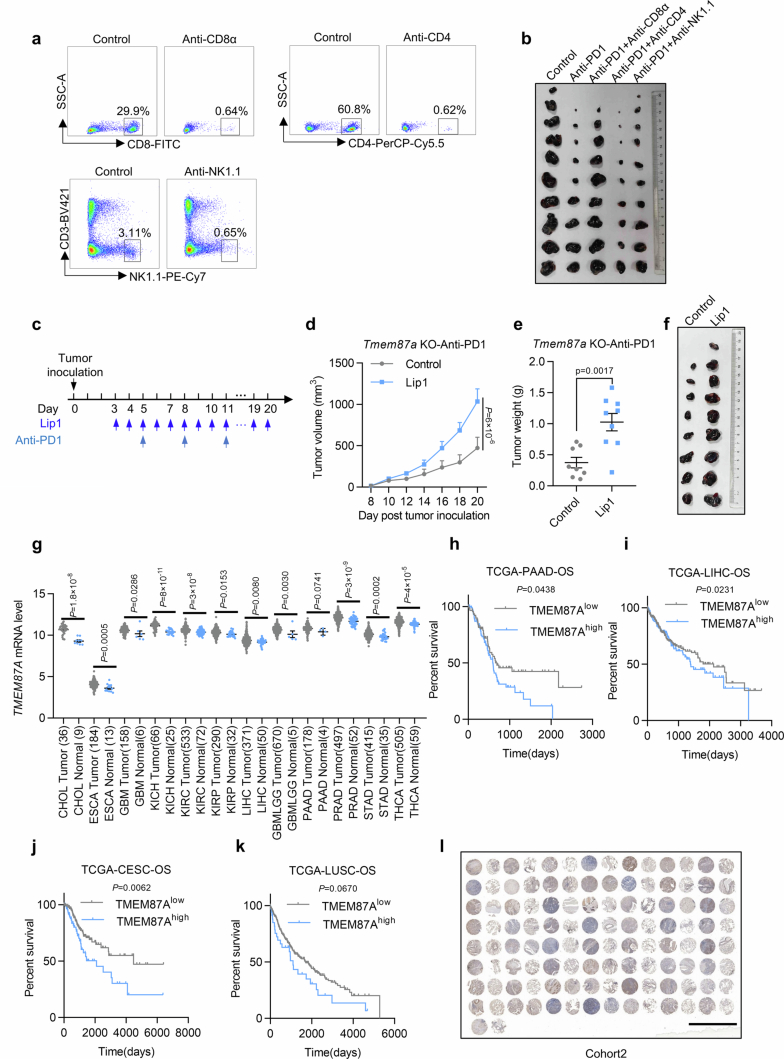
Ferroptosis inhibition attenuates the therapeutic efficacy of PD-1 blockade in TMEM87A deficient tumor and high level of TMEM87A correlates with poor prognosis. **a**, Representative flow cytometry analysis of CD8^+^ T cells, CD4^+^ T cell or NK cells subsets in splenocytes isolated from anti-CD8α, anti-CD4 or anti-NK1.1 antibody-treated mice. **b**, Representative image of *Tmem87a* KO B16F10 tumor isolated from C57BL/6 mice treated with anti-PD-1 (n = 9 mice), or anti-PD-1 combined with anti-CD8α (n = 9 mice), anti-CD4 (n = 9 mice) or anti-NK1.1 (n = 10 mice) antibody. **c- f**, Experimental design of *Tmem87a* KO B16F10 cells inoculated in C57BL/6 mice treated with anti-PD-1 antibody and liproxstatin-1 (Lip1) (**c**). Tumor volumes were monitored along the time (**d**). At the end, the weights (**e**) and representative image (**f**) of tumors isolated from these mice were shown. n = 8 (Control) or 9 (Lip1) mice per group). **g**, Data analysis of *TMEM87A* mRNA expression in human cancer tissues versus normal tissues from the TCGA database (CHOL, cholangiocarcinoma; ESCA, esophageal carcinoma; GBM, Glioblastoma multiforme; KICH, Kidney Chromophobe; KIRC, Kidney renal clear cell carcinoma; KIRP, Kidney renal papillary cell carcinoma; GBMLGG, Glioma; PRAD, prostate adenocarcinoma; STAD, stomach adenocarcinoma; THCA, Thyroid carcinoma). (n indicated in brackets in the figure). **h- k**, Kaplan-Meier analysis showing the overall survival (OS) of patients with PAAD (low, n = 89; high, n = 89) (**h**), LIHC (low, n = 221; high, n = 150) (**i**), CESC (low, n = 210; high, n = 93) (**j**) or LUSC (low, n = 464; high, n = 34) (**k**) with high or low TMEM87A expression from TCGA database. **l**, IHC staining of TMEM87A in human lung squamous cell carcinoma (LUSC) cohort (Cohort2) tissue microarray. Scale bar: 5 mm. All Data were shown as mean ± SEM. *P* values are determined by two-way ANOVA in (**d**), two-tailed t-test in (**e**), two-sided Wilcoxon rank-sum test in (**g**) and log-rank test in (**h**- **k**). Source data

To further confirm whether the above therapeutic efficacy is immune dependent, we depleted CD8^+^ T, CD4^+^ T or natural killer (NK) cells using specific antibodies in PD1 blockade-treated *Tmem87a*-KO B16F10 tumors (Fig. 6f and Extended Data Fig. 10a). As shown by tumor volume and weight, PD1 blockade significantly suppressed the growth of *Tmem87a*-KO tumors. Anti-CD8α but not anti-CD4 or anti-NK1.1 largely abolished the therapeutic effect of PD1 blockade (Fig. 6g,h and Extended Data Fig. 10b), suggesting that CD8^+^ T cells are the primary population mediating the therapeutic efficacy. We also investigated the effect of ferroptosis inhibitor liproxstatin 1 on PD1 blockade therapy in *Tmem87a*-KO tumors and found that liproxstatin 1 administration resulted in increased tumor volume and weight compared to the control group (Extended Data Fig. 10c–f). These data suggest that ferroptosis contributes to the therapeutic efficacy of PD1 blockade in Tmem87a-deficient tumors.

### High level of TMEM87A correlates with immunotherapy resistance

**Fig. 7:**
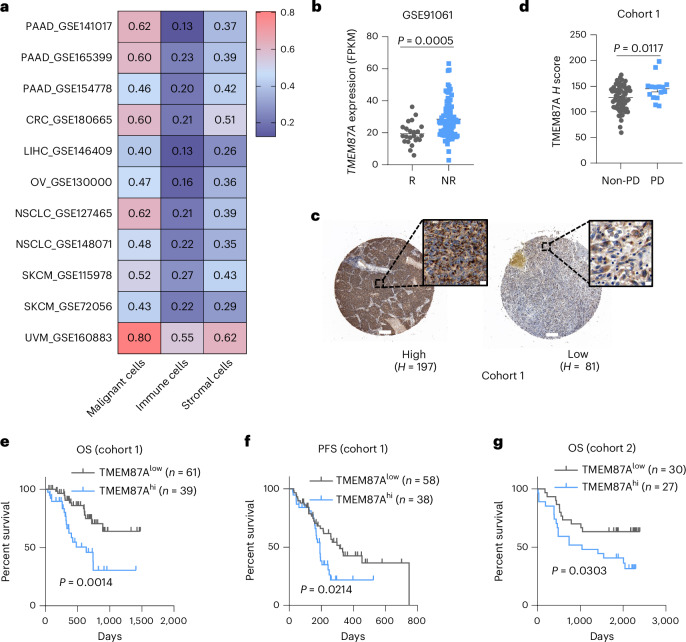
High level of TMEM87A correlates with immunotherapy resistance. **a**, *TMEM87A* mRNA expression in malignant cells, immune cells and stromal cells across different cancer scRNA-seq datasets from the Tumor Immune Single-Cell Hub database. PAAD, pancreatic adenocarcinoma; CRC, colorectal cancer; LIHC, liver HCC; OV, ovarian serous cystadenocarcinoma; NSCLC, non-small cell lung cancer; SKCM, skin cutaneous melanoma; UVM, uveal melanoma. **b**, Data analysis of tumoral TMEM87A expression between responders (R; *n* = 23 participants) and nonresponders (NR; *n* = 82 participants) with melanoma who received anti-PD1 immunotherapy. Data were obtained from the Gene Expression Omnibus (GSE91061). **c**, Representative images of TMEM87A IHC staining in human lung cancer specimens. Scale bar, 200 μm (default view) or 10 μm (zoomed-in view). **d**, Tumoral TMEM87A expression in immunotherapy-treated participants with progressive disease (PD; *n* = 70 participants) or nonprogressive disease (non-PD; *n* = 15 participants). **e**, Kaplan–Meier analysis showing the OS of participants with lung cancer with high (*n* = 61 participants) or low (*n* = 39 participants) TMEM87A expression. **f**, Kaplan–Meier analysis showing the PFS of participants with lung cancer with high (*n* = 58 participants) or low (*n* = 38 participants) TMEM87A expression. **g**, Kaplan–Meier analysis showing the OS of participants with lung cancer in another cohort with high (*n* = 30 participants) or low (*n* = 27 participants) TMEM87A expression. All data shown are the mean ± s.e.m. *P* values are indicated. Statistical analyses were conducted using a two-tailed *t*-test (**b**,**d**) or log-rank test (**e**–**g**). Source data

Lastly, we evaluated the clinical relevance of TMEM87A in the survival of persons with cancer, focusing on tumor types corresponding to our murine models. Analysis of publicly available scRNA-seq datasets revealed that *TMEM87A* mRNA was elevated in malignant cells compared to immune and stromal cell populations (Fig. 7a). Data from The Cancer Genome Atlas (TCGA) also showed a higher expression of *TMEM87A* mRNA in tumor compared to tumor-adjacent tissues (Extended Data Fig. 10g) and high levels of TMEM87A were negatively correlated with overall survival (OS) across multiple cancer types (Extended Data Fig. 10h–k). We then assessed the potential relevance of tumoral TMEM87A in cancer immunotherapy. In participants with melanoma treated with PD1 blockade, tumor tissues from responders exhibited lower *TMEM87A* expression compared to nonresponders (Fig. 7b). To further evaluate TMEM87A expression at the protein level, we performed a tissue microarray containing 100 biopsy specimens from participants with lung cancer who received immunotherapy together with or without chemotherapy and antiangiogenesis therapy (Supplementary Table 1). We found that TMEM87A protein was expressed from low to high levels in specimens and it predominantly localized near the nucleus within cancer cells (Fig. 7c). Higher expression of TMEM87A protein was found in the cohort whose disease progressed after receiving immunotherapy (Fig. 7d) and also correlated with shorter OS (Fig. 7e) and progression-free survival (PFS) (Fig. 7f). In another cohort consisting of 57 matched cancerous and normal tissue pairs from participants with lung squamous cell carcinoma (LUSC) (Supplementary Table 2), higher expression of TMEM87A again predicted worse OS (Fig. 7g and Extended Data Fig. 10l). These findings demonstrate that TMEM87A promotes tumor progression and confers immunotherapy resistance.

## Discussion

In addition to the PM, multiple subcellular membranes have been found to be susceptible to lipid peroxidation during ferroptosis, including the mitochondria^4,5,14^, lysosome^39^ and ER^6^. The Golgi apparatus, despite its direct association with the ER, had not been thoroughly investigated. Here we demonstrated that lipid peroxidation accumulated on the Golgi membrane in the early phase of ferroptosis. This immediate peroxidation of Golgi membrane lipids led to an elevation in Golgi pH, which could be completely reversed by Fer1. These results suggest that the Golgi pH elevation may serve as an early marker for ferroptosis initiation. Subsequently, when the Golgi pH was reduced or elevated, ferroptosis execution was promoted or suppressed, respectively, suggesting that the Golgi pH is also a regulatory mechanism for ferroptosis execution. In fact, emerging evidence has hinted that the Golgi apparatus is connected with ferroptosis^9,40^. A Golgi stress inducer brefeldin A was reported to trigger ferroptosis in Hela cells^40^. UBIAD1, a Golgi-localized antioxidant enzyme that regulates CoQ10 synthesis^41^, was shown to alleviate ferroptotic neuronal death in response to cerebral IRI^9^. In consistence with our findings, ferroptosis sensitization induced by acidosis (pH 6.5) was also observed in several human tumor cells, although it was explained by intracellular PUFA accumulation^42^. Considering that the Golgi apparatus maintains a unique resting pH to facilitate its efficient functions, including membrane trafficking, cargo selection, protein glycosylation and sorting^25,43,44^, it is worth further exploring whether these functions would be disturbed during ferroptosis occurrence.

We identified that the Golgi-localized TMEM87A has a critical role in maintaining Golgi pH homeostasis. TMEM87A was recently identified as a component of mechanoelectrical transduction pathways and was required for touch sensation in mice^22^. Ectopic expression of human TMEM87A in 293T cells presented both PM and Golgi localization^22^. Previous studies suggested that Golgi-resident TMEM87A might mediate the process of endosome-to-*trans*-Golgi network retrograde transport^19,45^. The structure analysis revealed that TMEM87A is similar to a canonical WNT transport protein WLS and might function in trafficking membrane-associated cargoes^20^. However, a recent study reported that TMEM87A might be a nonselective cation channel that mediates pH homeostasis of the Golgi^21^, which aligns with our results. The GYG motif in TMEM87A transmembrane domains (TMDs) was identified to be critical for its channel activity^21^. We also found that the TMEM87A-GYG-AAA mutant failed to maintain the Golgi pH, mediate ferroptosis resistance and support tumor growth. Therefore, TMEM87A-mediated Golgi pH homeostasis relies on its channel activity but the detailed mechanism by which TMEM87A is activated to regulate Golgi pH remains unclear. It is worth noting that a phosphatidylethanolamine (PE) was found to be bound in the TMD of human TMEM87A and might regulate its channel activity^21^. As PE containing PUFA chains will be preferentially peroxidized during ferroptosis^3,46^, it is possible that ferroptosis-induced lipid peroxidation may affect its channel activity. Future work could focus on identifying pharmacological inhibitors to block its channel activity. However, given its localization in the Golgi apparatus, this endeavor is likely to be challenging.

We found that TMEM87A ablation resulted in overacidification of the Golgi, which caused Fsp1–GFP condensation and sensitized tumor cells to ferroptosis. The phase separation of human FSP1 induced by iFSP1 has been reported to promote ferroptosis^33^. However, iFSP can only trigger human FSP1 but not mouse Fsp1 protein condensation^33^. Whether and under which conditions Fsp1 protein would undergo phase separation remains unknown, although it has been reported that IDR can drive phase separation in response to the alterations of pH or temperature^47,48^. Our findings indicated that TMEM87A ablation or an acidotic environment might be an initiating factor resulting in Fsp1 condensation. Tumoral ferroptosis induced by ferroptosis inducers or gene manipulations has been shown to benefit the inhibition of tumor growth and the activation of immune responses^18,49,50^. We identified that targeting TMEM87A could also promote tumoral ferroptosis, suppress tumor progression and enhance immunotherapy efficacy. However, it should be noted that ferroptosis in tumor-associated neutrophils could limit the activity of T cells^51^ and T cells themselves were also susceptible to ferroptosis^52^. Therefore, whether TMEM87A ablation affects the ferroptosis response and effector functions of these immune cells warrants further investigation.

In summary, we discovered that ferroptotic lipid peroxidation leads to an elevation in Golgi pH and identified that the Golgi-localized TMEM87A acts as a ferroptosis suppressor by buffering Golgi pH. Loss of TMEM87A can inhibit tumor growth and enhance antitumor CD8^+^ T cell immunity. Our findings hold promise for improving ICB therapy through targeting TMEM87A or modulating Golgi pH.

## Methods

### Ethics statement

All studies reported in this paper complied with the relevant ethics regulations. Tissue collection was approved by the institutional ethics board of Tongji Hospital of Tongji Medical College (TJ-IRB202412103). Informed consent was obtained from all human participants for the publication of the study results. The animal studies were conducted in accordance with the Institutional Animal Care and Use Committees (IACUCs) of Huazhong University of Science and Technology (IACUC no. 4293). For mouse tumor experiments, all tumor sizes and burdens were permitted by the IACUC. The maximal tumor burden of 2 cm^3^ permitted by the IACUC was not exceeded in this study.

### Key resources

Lists of resources for all antibody dilutions (Supplementary Table 3), reagents (Supplementary Table 4), cell lines and plasmids (Supplementary Table 5) and oligonucleotides (Supplementary Table 6) are provided in the Supplementary Information.

### CRISPR–Cas9-mediated gene KO

KO of *Tmem87a* or *Fsp1* in cell lines was performed with the CRISPR–Cas9 system. Single guide RNA (sgRNA) sequences were designed using the public online CRISPR design tool Synthego. sgRNAs were cloned into the LentiCRISPR-V2 vector. The LentiCRISPR plasmids together with psPAX2 and pMD2.G plasmids were transfected into 293T cells to produce lentivirus. Cancer cells were infected with lentivirus and then selected with puromycin for 3–4 days. Single-cell clones were sorted in 96-well plates for proliferation. The gene deletion in each cell clone was verified by immunoblotting.

*Tmem87a*-KO tumor cells that reexpressed Flag-tagged Tmem87a were constructed through the transient transfection of tumor cells with *Tmem87a* sgRNA plasmids, selection with puromycin and isolation of individual clones. The gene deletion in each cell clone was verified by immunoblotting to obtain *Tmem87a*-KO cells without puromycin resistance. The cDNA of Tmem87a was amplified by PCR. The Tmem87a-GYG-AAA mutant was generated by site-directed PCR mutagenesis using the WT Tmem87a template. The WT or mutant *Tmem87a* was cloned into the pLenti-CMV-3×Flag vector to produce lentivirus. Following lentiviral infection of *Tmem87a*-KO cells, puromycin selection was applied to ensure that surviving cells expressed either WT or mutant Tmem87a. The overexpression efficiency was verified by immunoblotting and localization was determined by immunofluorescence.

To obtain *Tmem87a* and *Acsl4* double-KO cells, *Tmem87a*-KO cells without puromycin resistance were infected by lentivirus expressing *Acsl4* sgRNAs, selected with puromycin and isolated to be individual clones. The deletion of Ascl4 in each cell clone was verified by immunoblotting. All primer sequences are listed in Supplementary Table 6.

### shRNA-mediated gene knockdown

Knockdown of *Tmem87a* in cell lines was performed with shRNAs. shRNA sequences were designed using the public online tool Sigma Predesigned. shRNAs were cloned into the pLKO.1 vector. Lentivirus production and cancer cell infection were performed similarly to the method described above. The knockdown efficiency was verified by immunoblotting or qPCR.

### Golgi pH measurements and image acquisition

Cancer cells were infected with lentiviral ratiometric reporters targeted to Golgi protein B4galt1 constructed on the basis of previously reported designs^24^ with SEP and mCherry.

For pH calibration experiments, pH calibration curves were generated as previously described^53^. In brief, cancer cells expressing B4galt1–mCherry–SEP were harvested and rinsed twice with one of the pH calibration curve buffers. The pH calibration curve buffers contained 125 mM KCl, 25 mM NaCl, 10 μM monensin and 25 mM HEPES (pH 7.5 or 7.0) or 25 mM MES (pH 7.0, 6.5, 6.0 or 5.5). Each buffer solution was adjusted to the appropriate final pH using 1 N NaOH or 1 N HCl. After rinsing, cells were resuspended in 200 μl of pH calibration curve buffer and analyzed immediately on a flow cytometer. The mean fluorescence intensity ratio of the SEP signal to mCherry signal for each pH calibration curve buffer was calculated and plotted to generate a pH calibration curve for further experiments in determining Golgi pH values. To determine Golgi pH values, cancer cells expressing B4galt1–mCherry–SEP were stimulated with BafA1 or NH_4_Cl for 1 h or RSL3 for the indicated time. Cells were harvested and resuspended in 200 μl of PBS and then analyzed immediately on a flow cytometer.

For dynamic imaging assays (Fig. 1e and Extended Data Fig. 1h), B16F10 cells expressing B4galt1–mCherry–SEP were seeded in glass-bottom dishes and stained with 1 µg ml^−1^ Hoechst 33342 for 30 min at 37 °C and then washed with PBS. Cells were stimulated with 2 μM RSL3 for 2 h or 1 μM BafA1 for 1 h, maintained in an incubator set at 37 °C with 5% CO_2_. The dynamic fluorescence images were acquired by a multimode laser confocal imaging system (with living cell workstation) (Leica) equipped with a ×63 (numerical aperture (NA): 1.20) CS2 Water MotCORR objective. Representative single-plane images were shown.

For other images showing Golgi pH (Fig. 1j and Extended Data Figs. 1f,g and 4d), B4galt1–mCherry–SEP-expressing cells seeded in glass-bottom dishes were treated with RSL3 or Fer1 for the indicated time. Subsequently, cells were stained with Hoechst 33342, washed and imaged in fresh medium. Images were acquired using an Olympus FV3000 laser scanning confocal microscope system equipped with a ×100 (NA: 1.45) silicone oil objective and photomultiplier tube detectors. Representative single-plane images were shown.

### Lipid peroxidation measurement

Cells that were treated with RSL3 for the indicated concentration and time were stained with 5 µM BODIPY 581/591 C11 for 30 min at 37 °C, washed and resuspended in 200 µl of PBS. Lipid peroxidation was assessed immediately on a flow cytometer. A minimum of 10,000 single cells were analyzed per sample.

For live-cell imaging of lipid peroxidation, cancer cells expressing B4galt1–BFP were seeded in glass-bottom dishes, treated with RSL3 and then stained with 5 µM BODIPY 581/591 C11, before washing and imaging in fresh medium. Images were acquired using an Olympus FV3000 confocal microscope with a ×100 (NA: 1.45) silicone oil objective. Quantification of mean fluorescence intensity of oxidized BODIPY C11 in Golgi was performed by ImageJ. The B4galt1–BFP^+^ area was selected as the region of interest (ROI) and the mean gray value of oxidized BODIPY C11 signal in the selected ROI was then calculated by the software.

### Immunofluorescence

For B4galt1–BFP location, B16F10 cells expressing B4galt1–BFP were seeded in glass-bottom dishes and treated with or without RSL3, followed by Tgn38 staining. Cells were fixed with 4% paraformaldehyde for 15 min and permeabilized with 0.1% Triton X-100 for 15 min. Subsequently, cells were washed with PBS, blocked in 5% goat serum and then incubated with anti-Tgn38 antibody at 4 °C overnight. After washing with PBS with Tween-20 (PBST), cells were incubated with anti-Rabbit IgG H + L (Alexa Fluor 488) for 2 h at room temperature, washed with PBST and imaged in fresh PBS.

For endogenous Tmem87a localization, B4galt1–GFP-expressing cells were stained with anti-Tmem87a antibody followed by incubation with ABflo 647-conjugated goat anti-rabbit IgG (H + L). For exogenous Tmem87a–Flag localization, cells expressing both B4galt1–GFP and Tmem87a–Flag were staining with anti-Flag antibody followed by incubation with anti-mouse IgG (H + L), F(ab′)2 Fragment (Alexa Fluor 647 conjugate). Nuclei were then counterstained with DAPI (5 µg ml^−1^). Eventually, cells were rinsed with PBST and imaged.

Images were acquired using an Olympus FV3000 confocal microscope with a ×100 (NA: 1.45) silicone oil objective. Representative single-plane images (Fig. 2b,c and Extended Data Fig. 1a) were shown. Colocalization analyses between B4galt1–BFP and Tgn38 were performed with ImageJ (version 1.54f) and analyzed using the line-scan plot profile that listed the signal gray value at different distances.

### Fsp1 imaging and fluorescence recovery after photobleaching (FRAP) assay

For Fsp1 imaging, cancer cells expressing Fsp1–GFP or Fsp1^ΔIDR1^–GFP were seeded in glass-bottom dishes, treated with 10% 1,6-HEX for 5 min (or without any treatment) and imaged using an Olympus FV3000 confocal microscope with a ×100 (NA: 1.45) silicone oil objective. Representative single-plane images were shown (Fig. 3c,e,i,k and Extended Data Fig. 7i).

For the FRAP assay, cancer cells expressing Fsp1–GFP were seeded in glass-bottom dishes and imaged to identify puncta before initiation of bleaching. Stimulation ROIs were set to cover the target puncta over multiple fields of view. After one baseline image was acquired, ROIs were bleached using a 488-nm laser line for 500 ms with 15% laser power. Images were collected for eight cycles with free-run interval postbleaching. Images were acquired using an Olympus FV3000 confocal microscope with a ×100 (NA: 1.45) silicone oil objective. Fluorescence intensity analysis was measured using ImageJ software.

### Transmission electron microscopy (TEM) analysis of Golgi morphology

To examine Golgi morphology at the ultrastructural level, samples were processed for TEM following standardized protocols. Briefly, cells were fixed in 2.5% glutaraldehyde in 0.1 M phosphate buffer, thoroughly washed and fixed again using 1% osmium tetroxide. Dehydration was performed twice using a graded ethanol series. Infiltration involves sequential treatment with acetone–epoxy resin mixtures (2:1, 1:1) and pure epoxy resin. Samples were then embedded in epoxy resin and cut into ultrathin sections. Finally, sections were double-stained with 2% uranyl acetate and lead citrate and examined under an FEI Tecnai G20 Twin TEM instrument.

### Cell viability assay

Cells were seeded in 96-well plates at 4 × 10^3^ cells per well and treated with reagents for the indicated concentration and time. Then, 10 µl of CCK-8 reagent was added to the culture medium and incubated for 4–6 h at 37 °C, 5% CO_2_. The absorbance at a wavelength of 450 nm was measured using a multimode microplate reader.

### Cell death assay

For cell death assays, cells were seeded in 24-well plates and treated with different reagents. Cells, including the suspended dying cells, were collected and resuspended in 200 μl of PBS containing 1 μg ml^−1^ propidium iodide (PI) for 15 min at room temperature. Cell death was assessed immediately on a flow cytometer. The PI^+^ population represented the percentage of dead cells.

### Immunoblotting

Cell pellets were collected and lysed by RIPA buffer supplemented with protease inhibitor cocktail. The protein concentration was quantified using a BCA protein assay kit. Except for protein samples to analyze Tmem87a, equal amounts of protein lysates were heated at 95 °C for 10 min. Protein samples were separated in 10% SDS–PAGE gels and transferred onto polyvinylidene fluoride membranes. Membranes were blocked in Tris-buffered saline with Tween-20 (TBST) containing 5% nonfat milk for 1 h at room temperature and incubated with primary antibodies at 4 °C overnight. The next day, Membranes were washed in TBST and incubated with horseradish-peroxidase-conjugated secondary antibodies for 1 h at room temperature. Immunoblots were imaged using a ChemiDoc imaging system. The antibodies used were listed in Supplementary Table 3.

### CoQ measurements

Cells for CoQ content measurements were harvested and frozen. Then, 200 μl of 96% ethanol containing 2,6-di-*tert*-butyl-4-methylphenol and 800 μl of 1-propanol were added to the frozen cells. The cell suspension was treated with five cycles of sonication and resting, frozen at −40 °C for 30 min and 4 °C for 10 min and then centrifuged at 15,000*g* for 15 min. The supernatant was dried and redissolved in ethanol (with internal standard CoQ10-D9) before performing ultrahigh-performance liquid chromatography–tandem mass spectrometry (UHPLC–MS/MS). Then, 5–5,000 ng ml^−1^ ubiquinone and ubiquinol were isometrically mixed with 200 ng ml^−1^ CoQ10-d9 to generate calibration curves. UHPLC–MS/MS analysis was performed using an Agilent 1290 Infinity II UHPLC system coupled to a 6470A triple-quadrupole MS instrument. Multiple reaction monitoring mode was applied to determine ubiquinone (880.7→197 for quantification and 880.7→95 for qualification) and ubiquinol (882.7→197 for quantification and 882.7→95 for qualification). The Agilent MassHunter software was used to acquire and analyze data. The concentration of oxidized and reduced CoQ was calculated on the basis of the calibration curve and normalized to protein concentration in each sample.

### Animals

Female WT C57BL/6JNifdc mice (aged 6–8 weeks) were obtained from Beijing Vital River Laboratory Animal Technology. C57BL/6-Tg (TcraTcrb) 1100Mjb/J mice were kindly provide by N. Wu (Tongji Medical College, Huazhong University of Science and Technology (HUST)). *Tmem87a*^f/+^, Alb^cre^ and *Tmem87a*^+/−^ mice were obtained from Shanghai Biomodel Organism Science and Technology Development. *Tmem87a* whole-body KO mice (*Tmem87a*^−/−^) were generated by crossing *Tmem87a*^+/−^ mice with *Tmem87a*^+/−^ mice. *Tmem87a*^f/f^ mice were generated by crossing *Tmem87a*^f/+^ mice with *Tmem87a*^f/+^ mice. The hepatocyte-specific *Tmem87a*-KO (*Tmem87a*^f/f^Alb^cre^) mice were generated by crossing *Tmem87a*^f/f^ with Alb^cre^ mice. The primers for genotyping are listed in Supplementary Table 6.

All animals were housed in the specific-pathogen-free facilities. Sex was not considered in the study design and analysis because this study was not designed to detect sex differences. For the spontaneous HCC model, only male mice were used. For other subcutaneous tumor models, female mice were used.

### Liver injury mouse models induced by ConA or anti-Fas antibody

For ConA-induced liver injury, 15 mg kg^−1^ ConA was injected into *Tmem87a*^f/f^Alb^cre^ mice and *Tmem87a*^f/f^ mice through the tail vein, while an equal volume of normal saline was injected as a control. Mice were killed 24 h after injection. For anti-Fas antibody-induced liver injury, 0.5 μg g^−1^ anti-Fas antibody was injected into *Tmem87a*^+/+^ mice and *Tmem87a*^−/−^ mice through the tail vein and mice were killed 5 h after injection. The blood of mice was collected to detect ALT and AST. Liver tissues were prepared into paraffin sections to perform hematoxylin and eosin (H&E) staining and the TUNEL assay or homogenized to perform immunoblots to detect the protein levels of Tmem87a, caspase 3 and cleaved Parp.

### Kidney IRI mice models

Bilateral kidney pedicles of *Tmem87a*^−/−^ mice were clamped using a hemostatic forceps for 45 min and then clamps were removed, leading to kidney IRI. The same operation or a sham operation (where mice were given laparotomy but the kidney pedicles were not clamped) was performed in WT mice. Liproxstatin 1 (10 mg kg^−1^) was intraperitoneally injected 1 h before clamping. After 24 h, mice were killed. The blood of mice was collected to detect CRE and BUN. Parts of the kidney tissues of mice were homogenized to detect the Tmem87a protein level through immunoblot analysis. Parts of the kidney tissues of mice were made into paraffin sections for H&E staining and immunohistochemistry (IHC) staining of 4-HNE.

### Subcutaneous tumor models

For subcutaneous tumor models, B16F10 cells (3 × 10^5^ cells per mouse), Panc02 cells (6 × 10^6^ cells per mouse) were suspended in PBS and subcutaneously injected into the right flank of WT C57BL/6 mice. CT26 cells (2 × 10^6^ cells per mouse) were suspended in PBS and subcutaneously injected into the right flank of BALB/c mice. Tumor volume was measured every 2 days when tumors were palpable using calipers and calculated as length × width^2^/2. At the end point, tumors of mice were stripped and weighed. Liproxstatin 1 (10 mg kg^−1^) was intraperitoneally injected into mice every day. Anti-PD1 antibody was intraperitoneally injected on days 5, 8 and 11 after tumor cell injection at a dose of 100 μg per mouse. Anti-CD8a antibody, anti-CD4 antibody and anti-NK1.1 antibody were intraperitoneally injected every 3 days started from day 4 after tumor cell injection at a dose of 100 μg per mouse.

For the preimmunization tumor model, freeze–thaw-treated or RSL3-treated B16F10 cells were obtained in vitro at first. For the freeze–thaw treatment, B16F10 cells underwent five complete freeze–thaw cycles consisting of snap-freezing in liquid nitrogen (5 min) followed by rapid thawing in a 37 °C water bath (5 min). For RSL3 treatment, B16F10 cells were exposed to 2 μM RSL3 for 24 h to induce cell death, with subsequent collection of detached dead cells. The freeze–thaw-treated or RSL3-treated B16F10 cells (5 × 10^5^ cells per mouse) and the WT or *Tmem87a*-KO B16F10 cells (3 × 10^5^ cells per mouse) were subcutaneously injected into the left flank of C57BL/6 mice. Then, 7 days after the immunization injection, normal B16F10 cells (3 × 10^5^ cells per mouse) were subcutaneously injected into the right flank. Tumor volumes in the right flank were measured every 2 days when tumors were palpable.

### Spontaneous HCC model

AKT–NRAS hydrodynamic tail-vein injection-induced spontaneous HCC was generated as previously described^54^. Briefly, 5 μg of RAS/pT3EF1a, 5 μg AKT/pT3EF1a and 0.67 μg of *Sleeping Beauty* transposase were diluted in 1.5 ml of 0.9% NaCl and hydrodynamically injected through the lateral tail vein into 4-week-old *Tmem87a*^f/f^ mice and *Tmem87a*^f/f^Alb^cre^ mice. Livers of mice were collected and weighed at 5 weeks after injection.

For liver-specific inducible *Tmem87a* KO, 7 days after hydrodynamic injection of RAS/pT3EF1a, AKT/pT3EF1a and *Sleeping Beauty* transposase into *Tmem87a*^f/f^ mice, 1 × 10^11^ viral genome copies of AAV8-hTBG-iCre virus or AAV8-hTBG-GFP control virus (AAV8-control) diluted in 100 μl of saline were injected through the lateral tail vein. Mice were killed on day 35 and livers were collected to verify *Tmem87a* KO by immunoblotting.

### scRNA-seq

*Tmem87a*-KO B16F10 tumors reexpressing empty vector or Tmem87a–Flag were dissected from C57BL/6 mice and single-cell suspensions were obtained. CD45^+^ cells were extracted by flow cytometric sorting. The cell suspension was barcoded and RNA from the barcoded cells was reverse-transcribed to construct sequencing libraries (10x Genomics). Sequencing was performed with Illumina NovaSeq.

Sequencing reads were demultiplexed and aligned to the reference genome by Cell Ranger. Analyses were performed using Cell Ranger and Seurat. Unique molecule identifiers were counted to construct digital expression matrices. Quality control filtering was applied to retain genes detected in at least five cells and cells expressing a minimum of 300 genes. The ‘find variable features’ function, ‘normalize data’ function, principal component analysis (PCA) and uniform manifold approximation and projection (UMAP) dimensionality reduction were performed to identify 14 distinct clusters. Differential expression analysis was performed between each cluster and all other cells using a Wilcoxon rank-sum test. The ‘add module score’ function in Seurat was used to calculate the module scores of the public cell state gene signatures. Features for the 14 immune cell clusters and CD8^+^ T cell activation, migration and effector function are summarized in Supplementary Tables 7 and 8.

### Flow cytometry analysis

Tumor or spleens of different groups from mice were collected at the end point, ground using a grinder and prepared into a single-cell suspension using 70-μm cell strainers. Single-cell suspensions were stained with Zombie near-infrared (NIR) dye, blocked by CD16/CD32 and then labeled with fluorochrome-conjugated anti-CD45, anti-CD3, anti-CD4, anti-CD8a, anti-CD25, anti-NK1.1, anti-CD11b, anti-Ly6G, anti-Ly6C, anti-F4/80 and anti-CD11c. For transcript staining, cells were fixed and permeabilized using the transcription factor buffer set and stained with anti-FOXP3. For cytokine detection, cells were suspended in complete culture medium and stimulated with monensin (1:1,000), ionomycin (1 μg ml^−1^), brefeldin A (1:1,000) and phorbol 12-myristate 13-acetate (20 ng ml^−1^) at 37 °C for 4 h in an incubator. Cells were washed, stained with Zombie NIR dye and then labeled with anti-CD45, anti-CD3, anti-CD4 and anti-CD8a. Cells were fixed and permeabilized using fixation buffer and perm/wash buffer, respectively, and then stained with anti-IFNγ and anti-TNF diluted in perm/wash buffer. Finally, cells were resuspended in 500 μl of PBS and assessed immediately on a flow cytometer. The flow cytometry data were analyzed by FlowJo software.

### IHC

IHC was performed using the IHC prep and detect kit according to the manufacturer’s instructions. For staining of 4-HNE in mouse kidneys and staining of TMEM87A in human lung carcinoma in tissue microarrays, the antibodies listed in Supplementary Table 3 were used. Three fields of each tumor sample were selected to determine the *H* score, which was automatically measured by the FIJI IHC Profiler to get the percentage of high positive, positive, low positive and negative. The *H* score was calculated as 1 × (% low positive) + 2 × (% positive) + 3 × (% high positive). According to *H* score ranking, tumor samples were divided into groups with high and low TMEM87A expression.

### OT-I and tumor cell coculture

The spleen and lymph nodes were excised from C57BL/6-Tg (TcraTcrb) 1100Mjb/J mice (OT-I mice) and prepared into a single-cell suspension using 70-μm cell strainers. OT-I CD8^+^ T cells were expanded and activated in culture medium containing 10% FBS, 5 μg ml^−1^ OVA_257__–264_ peptide, β-mercaptoethanol (1:1,000) and penicillin–streptomycin (1:100). Then, 10 ng ml^−1^ mouse recombinant IL-2 was added on the second day and cells were harvested on the fourth day.

WT and *Tmem87a*-KO B16F10 cells were stably transfected with OVA–Luc-expressing lentivirus and selected with blasticidin for 1–2 weeks. Before coculture with OT-I cells, the luciferase intensity in WT and *Tmem87a*-KO tumor cells was quantified to ensure comparable transfection efficiency. OVA^+^Luc^+^ tumor cells were seeded into 96-well plates and incubated overnight. The next day, different proportions of OT-I CD8^+^ T cells were added for coculture. After the indicated time, the luciferase intensity of adherent tumor cells was quantified using Nano-Glo luciferase assay reagent to represent the viability of tumor cells.

### Human samples

Tissue specimens of clinical participants with lung cancer contained in two independent tissue microarrays were collected at Tongji Hospital (HUST). One tissue microarray cohort consisted of 100 evaluable cores with the pathologic diagnosis of lung carcinoma. Participants received immunotherapy, with or without chemotherapy and antiangiogenesis therapy. Data included the OS of 100 participants and disease-free survival of 96 participants. The second cohort comprised 114 cores for matched cancer tissue and normal tissue pairs of 57 participants with LUSC, accompanied by the OS. All samples and information were collected with informed consent of the participants. The sex distribution and characteristics of the participants are detailed in Supplementary Tables 1 and 2. Race, ethnicity and other socially relevant categorizations were not considered or collected in this study. Therefore, such variables were not used as proxies.

### Statistics and reproducibility

Statistical analyses were performed using GraphPad Prism8 software. All quantitative data are presented as the mean ± s.e.m. The comparisons were analyzed using an unpaired two-tailed Student’s *t*-test or analysis of variance (ANOVA). Data distribution was assumed to be normal when using *t*-tests but this was not formally tested. Individual data points are shown. Kaplan–Meier curves were generated by log-rank tests. Exact *P* values are indicated in the related figures. The experiments in this study were set up using 3–10 samples per independent group, condition or repeat. Biological replications and statistics are indicated in the legends. The fluorescence imaging and immunoblotting experiments are shown as representative results from three independent experiments with similar results. No statistical methods were used to predetermine sample sizes but our sample sizes are similar to those reported in previous publications^13,15,55^. No data were excluded from the analyses. For animal experiments, all mice were matched for sex and age before randomizing into different groups, whereas the other experiments were not randomized. Data collection and analysis were not performed blind to the conditions of the experiments, except for the IHC score evaluation.

### Reporting summary

Further information on research design is available in the Nature Portfolio Reporting Summary linked to this article.

## Supplementary information

Supplementary Information Supplementary Fig. 1. Gating strategies used in FACS analysis.

Reporting Summary

Supplementary Tables 1–8 Supplementary Tables 1–8.

## Source data

Source Data Figs. 1–7 and Extended Data Figs. 1–10 Statistical source data.

Source Data Fig. 2 and Extended Data Figs. 3–8 Unprocessed western blots.

## Data Availability

The raw scRNA-seq data generated in this study were deposited to the Genome Sequence Archive under accession code CRA025122. Data for TMEM87A expression and correlated small molecules (Fig. 2a and Extended Data Fig. 2h) are publicly available from the CTRP database (https://portals.broadinstitute.org/ctrp/). The human data (Fig. 7a or Extended Data Fig. 10g–k) were derived from the Tumor Immune Single-Cell Hub database (http://tisch.comp-genomics.org/search-gene/) or TCGA of the National Cancer Institute (https://xenabrowser.net/datapages/). Previously published scRNA-seq data that were reanalyzed here (Fig. 7b) are available from the GEO under accession code GSE91061 (ref. ^56^). Source data are provided with this paper.

## Code availability

No custom code was generated for this study.
