# Supplementary material for: TMEM87A suppresses ferroptosis and increases cancer immunotherapy resistance by maintaining the Golgi apparatus pH homeostasis
Source: Nat Cancer. 2026 Apr 21;7(5):823–39. doi: 10.1038/s43018-026-01156-9 (PMC13221295; doi:10.1038/s43018-026-01156-9)

nature cancer

Supplementary information

https://doi.org/10.1038/s43018-026-01156-9

TMEM87A suppresses ferroptosis and increases cancer immunotherapy resistance by maintaining the Golgi apparatus pH homeostasis

In the format provided by the authors and unedited

Supplementary Information

Supplementary Figure 1. Flow cytometry gating strategies for tumor-infiltrating T cells

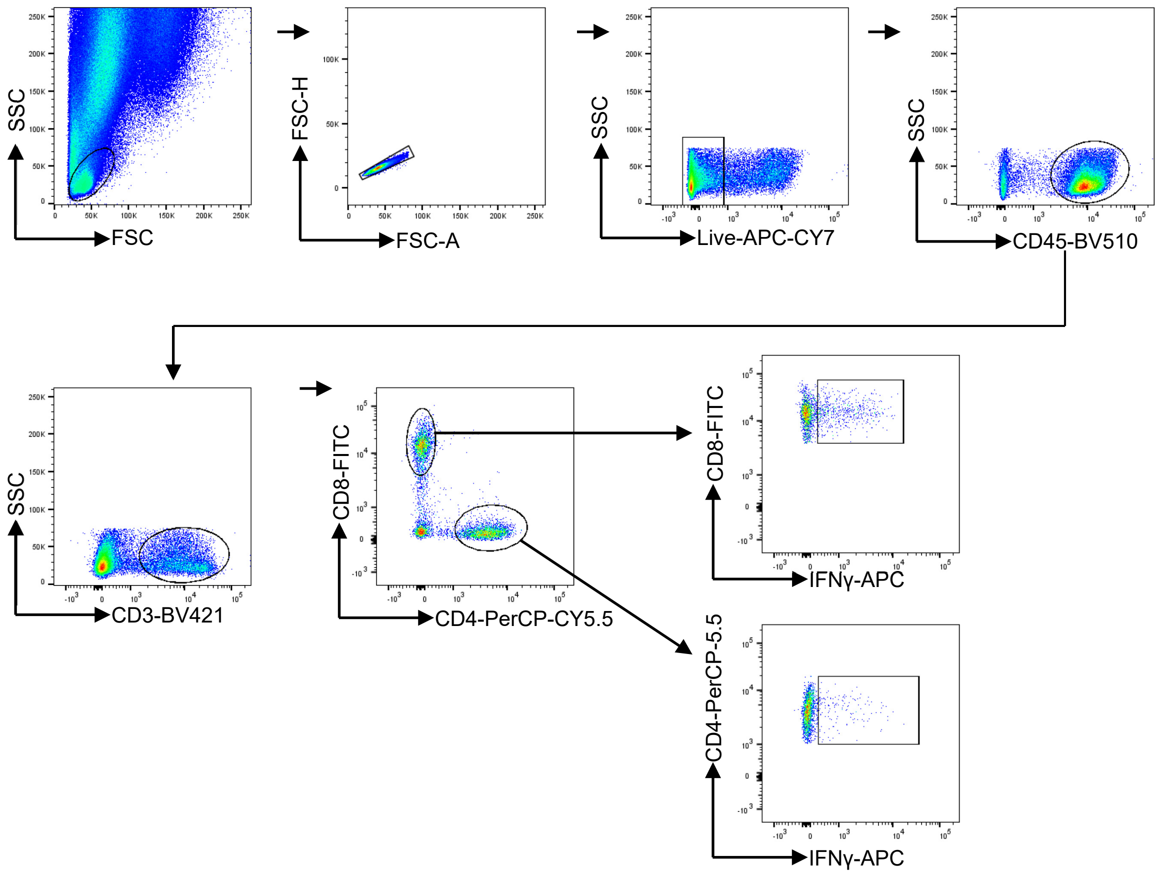

Supplement: Supplementary file 1 — Supplementary Fig. 1. Gating strategies used in FACS analysis. [file 43018_2026_1156_MOESM1_ESM.pdf]
