# Supplementary material for: TMEM87A suppresses ferroptosis and increases cancer immunotherapy resistance by maintaining the Golgi apparatus pH homeostasis
Source: Nat Cancer. 2026 Apr 21;7(5):823–39. doi: 10.1038/s43018-026-01156-9 (PMC13221295; doi:10.1038/s43018-026-01156-9)
Supplement: Supplementary file 2 — Reporting Summary [file 43018_2026_1156_MOESM2_ESM.pdf]

nature portfolio

Corresponding author(s): Weimin Wang

Last updated by author(s): Feb 26, 2026

Reporting Summary

Nature Portfolio wishes to improve the reproducibility of the work that we publish. This form provides structure for consistency and transparency in reporting. For further information on Nature Portfolio policies, see our Editorial Policies and the Editorial Policy Checklist.

Statistics

For all statistical analyses, confirm that the following items are present in the figure legend, table legend, main text, or Methods section.

n/a	Confirmed	
☐	☒	The exact sample size (*n*) for each experimental group/condition, given as a discrete number and unit of measurement
☐	☒	A statement on whether measurements were taken from distinct samples or whether the same sample was measured repeatedly
☐	☒	The statistical test(s) used AND whether they are one- or two-sided<br>*Only common tests should be described solely by name; describe more complex techniques in the Methods section.*
☒	☐	A description of all covariates tested
☐	☒	A description of any assumptions or corrections, such as tests of normality and adjustment for multiple comparisons
☐	☒	A full description of the statistical parameters including central tendency (e.g. means) or other basic estimates (e.g. regression coefficient) AND variation (e.g. standard deviation) or associated estimates of uncertainty (e.g. confidence intervals)
☐	☒	For null hypothesis testing, the test statistic (e.g. *F*, *t*, *r*) with confidence intervals, effect sizes, degrees of freedom and *P* value noted<br>*Give P values as exact values whenever suitable.*
☒	☐	For Bayesian analysis, information on the choice of priors and Markov chain Monte Carlo settings
☒	☐	For hierarchical and complex designs, identification of the appropriate level for tests and full reporting of outcomes
☐	☒	Estimates of effect sizes (e.g. Cohen's *d*, Pearson's *r*), indicating how they were calculated

Our web collection on statistics for biologists contains articles on many of the points above.

Software and code

Policy information about availability of computer code

Data collection	Flow cytometer BD LSRFortessa (BD) , BD verse (BD) and Thermo Attune NxT (Thermo Fisher Scientific) were used to run cell samples and BD FACSuite v1.0.6 and AttuneTM Cytometric Software v5.1.1 were used to collect data; QuantStudio® 3 Real-Time PCR System (Thermo Fisher Scientific) was used to run qPCR; ChemiDoc Imaging System (Tanon-5200Multi) was used for collecting data from immunoblotting experiments; Microplate reader (BioTek Synergy H1) was used for the assays requiring absorbency and luminescence quantification; Slide-scanning platform (3DHISTECH) was used for automated slide-scanning; OLYMPUS FV3000 microscope was used for collecting microscopy images. Single-cell sequencing was conducted using the Illumina NovaSeq platform(10x gemomics).
Data analysis	FlowJo software version 10 was used for FACS data analysis. QuantStudio Design and Analysis software v1.5.1 was for qPCR data analysis; Gen5 CHS 3.04 software was used for quantification of absorbency and luminescence data; Tanon AllDox-X software (v2.2.1) was used for analysis of immunoblotting results; CaseViewer2.4 software (3DHISTECH) was for analysis of IHC images; FLOWVIEW FV31S-SW software and ImageJ software were used for analysis of Immunofluorescence microscopy images; The Agilent MassHunter software (version B.08.00, Agilent) was used to extract and analyze mass spectrometry data. Single-cell sequencing data were analyzed using the Cell Ranger pipeline and the Seurat toolkit.GraphPad Prism version 8 was used for statistical analysis and P values calculation.

For manuscripts utilizing custom algorithms or software that are central to the research but not yet described in published literature, software must be made available to editors and reviewers. We strongly encourage code deposition in a community repository (e.g. GitHub). See the Nature Portfolio guidelines for submitting code & software for further information.

Data

Policy information about availability of data

All manuscripts must include a data availability statement. This statement should provide the following information, where applicable:

- Accession codes, unique identifiers, or web links for publicly available datasets
- A description of any restrictions on data availability
- For clinical datasets or third party data, please ensure that the statement adheres to our policy

The raw scRNA-seq data generated in this study have been deposited in the Genome Sequence Archive under accession codes CRA025122 (https://ngdc.cncb.ac.cn/gsa/browse/CRA025122). Data for TMEM87A expression and correlated small molecules (Fig. 2a and Extended Data Fig. 2h) are publicly available from CTRP databases (https://portals.broadinstitute.org/ctrp/). The human data (Fig. 7a or Extended Data Fig. 10g- k) were derived from TISCH database (http://tisch.comp-genomics.org/search-gene/) or the Cancer Genome Atlas Program (TCGA) of National Cancer Institute (https://xenabrowser.net/datapages/). Previously published scRNA-seq data that were re-analysed here (Fig. 7b) are available under accession code GSE91061. Source data are provided with this paper.

Research involving human participants, their data, or biological material

Policy information about studies with human participants or human data. See also policy information about sex, gender (identity/presentation), and sexual orientation and race, ethnicity and racism.

Reporting on sex and gender	Tumor tissue specimens were from the patients with lung cancer. The patients included both men and women, aged 25-76 years. The relevant information was provided in Supplementary Table S1 and Table S2.
Reporting on race, ethnicity, or other socially relevant groupings	The race, ethnicity or other socially relevant groupings were not considered for this study.
Population characteristics	The detailed clinical features of patients with lung cancer were provided in Supplementary Table S1 and Table S2.
Recruitment	Patients with lung cancer were diagnosed or received immunotherapy in Tongji Hospital of Huazhong University of Science and Technology.
Ethics oversight	The study was approved by the institutional ethics board of Tongji Hospital of Huazhong University of Science and Technology. The informed consent was obtained from all human participants for the publication of the study results.

Note that full information on the approval of the study protocol must also be provided in the manuscript.

Field-specific reporting

Please select the one below that is the best fit for your research. If you are not sure, read the appropriate sections before making your selection.

☒ Life sciences ☐ Behavioural & social sciences ☐ Ecological, evolutionary & environmental sciences

For a reference copy of the document with all sections, see nature.com/documents/nr-reporting-summary-flat.pdf

Life sciences study design

All studies must disclose on these points even when the disclosure is negative.

Sample size	No statistical method was used to calculate sample size. Sample sizes were chosen based on our previous experiences (Nature. 2019;569(7755):270-274; Nat Commun. 2023 Aug 8;14(1):4758; Cell Metab. 2024 Apr 2;36(4):822-838), which is sufficient to generate statistically significant results. For most of the in vitro experiments, at least three biological replicates were achieved for statistics. For in vivo experiments, a simple size of n = 5-10 mice per experimental group was used.
Data exclusions	No data was excluded.
Replication	All experiments were performed independently at least two or three times with similar results. All replication attempts were successful.
Randomization	For in vivo experiments, age and sex matched animals were firstly inoculated with tumor cells and then randomly assigned into different treatment groups. The initial tumor burden was similar between the treatment and control groups. For in vitro experiments, cells were equally distributed into culture dishes and followed by different treatments with replications, no randomization was required.
Blinding	Data collection and analysis were not performed blind to the conditions of the experiments, except for the IHC score evaluation.

Reporting for specific materials, systems and methods

We require information from authors about some types of materials, experimental systems and methods used in many studies. Here, indicate whether each material, system or method listed is relevant to your study. If you are not sure if a list item applies to your research, read the appropriate section before selecting a response.

Materials & experimental systems

n/a	Involved in the study
☐	☒ Antibodies
☐	☒ Eukaryotic cell lines
☒	☐ Palaeontology and archaeology
☐	☒ Animals and other organisms
☒	☐ Clinical data
☒	☐ Dual use research of concern
☒	☐ Plants

Methods

n/a	Involved in the study
☒	☐ ChIP-seq
☐	☒ Flow cytometry
☒	☐ MRI-based neuroimaging

Antibodies

Antibodies used

anti-mouse-PD-1 (Clone: RMP1-14, BE0146, Bio X Cell, 100µg/mouse),
anti-mouse CD8α-InVivo (clone: 2.43, A2102, Selleck, 100µg/mouse),
anti-mouse CD4-InVivo (clone: GK1.5; A2101, Selleck, 100µg/mouse),
anti-mouse NK1.1-InVivo (clone: PK136; A2114, Selleck, 50µg/mouse),
anti-TMEM87a (Sigma, HPA018104, 1:2000),
anti-Flag (Sigma, 1804, 1:2000),
anti-ACSL4 (Santa Cruz, sc365230, 1:2000),
anti-GAPDH (Proteintech, 60004, 1:5000),
anti-GPX4 (Proteintech, 67763-1-Ig, 1:1000),
anti-FSP1 (Proteintech, 20886-1-Ap, 1:2000),
anti-Caspase3 (Santa Cruz, sc56053, 1:500) ,
anti-Cleaved PARP(CST,5625,1:1000),
anti-TGN38 (CST, 85181, 1:500)
Goat anti-Rabbit IgG (H+L) Secondary Antibody, HRP (ThermoFisher Scientific, 31460, 1:10000) ,
Goat anti-Mouse IgG (H+L) Secondary Antibody, HRP (ThermoFisher Scientific, 31430, 1:10000),
Goat anti-Rabbit IgG H&L (Alexa Fluor® 488) (Abcam, ab150077, 1:500),
Goat anti-mouse IgG (H+L), F(ab')2 Fragment (Alexa Fluor® 647 Conjugate) (CST, 4410S, 1:500),
anti-4HNE (JaICA, HNEJ-2, 1:200),
anti-CD45 (Clone :30-F11, 103138, Brilliant Violet 510, Biolegend, 1:100),
anti-CD3e (Clone: 145-2C11, 562600, BV421, BD Biosciences, 1:100),
anti-CD90 (Clone: 53-2.1, 140319, BV510, Biolegend, 1:100),
anti-CD4 (Clone: GK1.5, 100433, PerCP/Cyanine5.5, Biolegend, 1:100),
anti-CD8 (Clone: 53-6.7, 100706, FITC, Biolegend, 1:100),
anti-IFNγ (Clone: XMG1.2, 505810, APC, Biolegend, 1:100),
anti-CD25 (Clone: PC61,102012, APC, Biolegend, 1:100),
anti-NK1.1 (Clone: PK136, 108713, PE/Cyanine7, Biolegend, 1:100),
anti-CD11b (Clone: M1/70, 101205, FITC, Biolegend, 1:100),
anti-CD11c (Clone: N418, 558079, PE/Cyanine7, BD Biosciences, 1:100),
anti-Ly6G (Clone: 1A8, 127613, APC, Biolegend, 1:100),
anti-Ly6C (Clone: HK1.4, 128007, PE, Biolegend, 1:100),
anti-F4/80 (Clone: T45-2342, 746070, BB700, BD Biosciences, 1:100).

Validation

All antibodies are commercial available and validated on the manufacturers website as listed below. Information about usage, validation and citations are available from the product page.
anti-mouse-PD-1: https://bioxcell.com/invivomab-anti-mouse-pd-1-cd279-be0146
anti-mouse CD8α-InVivo: https://www.selleck.cn/products/anti-mouse-cd8a-invivo.html
anti-mouse CD4-InVivo: https://www.selleck.cn/products/anti-mouse-cd4-invivo.html
anti-mouse NK1.1-InVivo: https://www.selleck.cn/products/anti-mouse-nk1-1-invivo.html
anti-TMEM87a: https://www.sigmaaldrich.cn/CN/en/product/sigma/hpa018104
anti-Flag: https://www.sigmaaldrich.cn/CN/en/product/sigma/f1804
anti-ACSL4: https://www.scbt.com/p/acsl4-antibody-f-4
anti-GAPDH: https://www.ptgcn.com/products/GAPDH-Antibody-60004-1-Ig.htm
anti-GPX4: https://ptgcn.com/products/GPX4-Antibody-67763-1-Ig.htm
anti-FSP1: https://www.ptgcn.com/products/AIFM2-Antibody-20886-1-AP.htm
anti-Caspase3:https://www.scbt.com/p/caspase-3-antibody-31a1067
anti-Cleaved PARP:https://www.cellsignal.com/products/primary-antibodies/cleaved-parp-asp214-d64e10-xp-rabbit-mab/5625
anti-TGN38:https://www.cellsignal.com/products/primary-antibodies/tgoln2-tgn38-e6b6a-rabbit-mab/85181
Goat anti-Rabbit IgG (H+L) Secondary Antibody, HRP: https://www.thermofisher.cn/cn/zh/antibody/product/Goat-anti-Rabbit-IgG-H-L-Secondary-Antibody-Polyclonal/31460
Goat anti-Mouse IgG (H+L) Secondary Antibody, HRP: https://www.thermofisher.cn/cn/zh/antibody/product/Goat-anti-Mouse-IgG-H-L-Secondary-Antibody-Polyclonal/31430
Goat anti-Rabbit IgG H&L (Alexa Fluor® 488): https://www.abcam.cn/products/secondary-antibodies/goat-rabbit-igg-hl-alexa-fluor-488-ab150077.html
Goat anti-mouse IgG (H+L), F(ab')2 Fragment (Alexa Fluor® 647 Conjugate): https://www.cellsignal.com/products/secondary-

antibodies/anti-mouse-igg-h-l-f-ab-2-fragment-alexa-fluor-647-conjugate/4410
anti-4HNE: https://www.jaica.com/e/products_lipid_4hne_ab.html
anti-CD45: https://www.biolegend.com/en-us/products/brilliant-violet-510-anti-mouse-cd45-antibody-7995
anti-CD3e: https://www.bdbiosciences.com/zh-cn/products/reagents/flow-cytometry-reagents/research-reagents/single-color-antibodies-ruo/bv421-hamster-anti-mouse-cd3e.562600?tab=product_details
anti-CD90: https://www.biolegend.com/en-us/products/brilliant-violet-510-anti-mouse-cd902-thy12-antibody-7999
anti-CD4: https://www.biolegend.com/en-us/products/percp-cyanine5-5-anti-mouse-cd4-antibody-4220
anti-CD8: https://www.biolegend.com/en-us/products/fitc-anti-mouse-cd8a-antibody-153
anti-IFNγ: https://www.biolegend.com/en-us/products/apc-anti-mouse-ifn-gamma-antibody-993
anti-CD25: https://www.biolegend.com/en-us/products/apc-anti-mouse-cd25-antibody-420
anti-NK1.1: https://www.biolegend.com/en-us/products/pe-cyanine7-anti-mouse-nk-1-1-antibody-2840
anti-CD11b: https://www.biolegend.com/en-us/products/fitc-anti-mouse-human-cd11b-antibody-347
anti-CD11c:https://www.bdbiosciences.com/zh-cn/products/reagents/flow-cytometry-reagents/research-reagents/single-color-antibodies-ruo/pe-cy-7-hamster-anti-mouse-cd11c.558079?tab=product_details
anti-Ly6G: https://www.biolegend.com/en-us/products/apc-anti-mouse-ly-6g-antibody-6115
anti-Ly6C: https://www.biolegend.com/en-us/products/pe-anti-mouse-ly-6c-antibody-4904
anti-F4/80: https://www.bdbiosciences.com/zh-cn/products/reagents/flow-cytometry-reagents/research-reagents/single-color-antibodies-ruo/bb700-rat-anti-mouse-f4-80.746070?tab=product_details

Eukaryotic cell lines

Policy information about cell lines and Sex and Gender in Research

Cell line source(s)	B16F10(CRL-6475), CT26.WT(CRL-2638) and 293T(CRL-3216) cell lines were from American Type Culture Collection (ATCC). Hepa1-6(SCSP-512) cell line were from the China Center for Type Culture Collection (CCTCC). Panc02(1101MOU-PUMC000446) cell line was was obtained from the Cell Resource Center, Peking Union Medical College (http://cellresource.cn). SW48 originating from ATCC was kindly provided by Dr. Zheng Wang (Huazhong University of Science and Technology).
Authentication	Hepa1-6 cell line was authenticated using the short tandem repeat analysis. It was done by a PCR-based strategy to target 17 STR loci and the result showed that the cell line matched with Hepa1-6 in Cellosaurus database. All other cell lines were not authenticated.
Mycoplasma contamination	All cell lines in our laboratory are routinely tested for mycoplasma contamination using a PCR-based method. And the cells used in this study are negative for mycoplasma.
Commonly misidentified lines (See ICLAC register)	No cell line used in this study is listed in ICLAC database.

Animals and other research organisms

Policy information about studies involving animals; ARRIVE guidelines recommended for reporting animal research, and Sex and Gender in Research

Laboratory animals	Female wild-type C57BL/6JNifdc mice (aged 6–8 weeks) were obtained from Beijing Vital River Laboratory Animal Technology Co., Ltd. C57BL/6-Tg (TcraTcrb) 1100Mjb/J mice were kindly provide by Dr. Ning Wu (Tongji Medical college, Huazhong University of Science and Technology). Tmem87a f/+, Albcre and Tmem87a+/- mice (aged 8 weeks) were obtained from Shanghai Biomodel Organism Science & Technology Development Co., Ltd.<br>Tmem87a constitutive KO mice (Tmem87a-/-) were generated by crossing Tmem87a+/- mice with Tmem87a+/- mice.<br>Tmem87a f/f mice were generated by crossing Tmem87a f/+ mice with Tmem87a f/+ mice.<br>The hepatocyte-specific Tmem87a knockout (TMEM87af/f Albcre) mice were generated by crossing Tmem87af/f with Albcre mice.<br>All animals were housed under specific pathogen-free conditions in groups of 5 mice per cage, and maintained in a humidity-controlled environment with a 12 h light/dark cycle at a temperature of 22-25°C.
Wild animals	The study did not involve wild animals.
Reporting on sex	For spontaneous hepatocellular carcinoma model, only male mice were used; For other subcutaneous tumor models, female mice were used.
Field-collected samples	The study did not involve samples collected from field.
Ethics oversight	Animal studies were conducted in accordance with the Institutional Animal Care and Use Committees and Institutional Review Board of School of Basic Medicine, Tongji Medical College, Huazhong University of Science and Technology. All tumor sizes were permitted by the IACUC of Huazhong University of Science and Technology (HUST). Maximal tumor burden of 2cm3 permitted by the IACUC was not exceeded in this study.

Note that full information on the approval of the study protocol must also be provided in the manuscript.

Plants

Seed stocks	N/A
Novel plant genotypes	N/A
Authentication	N/A

Flow Cytometry

Plots

Confirm that:

☒ The axis labels state the marker and fluorochrome used (e.g. CD4-FITC).

☒ The axis scales are clearly visible. Include numbers along axes only for bottom left plot of group (a 'group' is an analysis of identical markers).

☒ All plots are contour plots with outliers or pseudocolor plots.

☒ A numerical value for number of cells or percentage (with statistics) is provided.

Methodology

Sample preparation

For cell death assays, cells were seeded in 24-well plates at 3×104 cells/well and treated with different reagents for the indicated concentration and time. Cells, including the suspended dying cells, were collected and resuspended in 200 µl PBS containing 1 µg/ml Propidium Iodide (PI) for 15 min at room temperature. Cell death was assessed immediately on a flow cytometer (BD FACSVerse). A minimum of 10,000 single cells were analyzed per sample. PI+ population represented the percentage of dead cells.

For intracellular lipid peroxidation analysis, cells were seeded in 24-well plates at 8×104 cells/well and incubated overnight. The next day, cells were treated with reagents for the indicated concentration and time, then washed and harvested by trypsinization. Cells were stained with 5 µM BODIPYTM 581/591 C11 (D3861, Thermo Fisher Scientific) for 30 minutes at 37 ° C, washed and resuspended in 200 µl PBS. Lipid peroxidation was assessed immediately on a flow cytometer (BD FACSVerse). A minimum of 10,000 single cells were analyzed per sample.

For Golgi pH experiments, cancer cells expressing B4galt1-mCherry-SEpHluorin were plated in 24-well plates at 40,000 cells/well, and stimulated with Bafilomycin A1 (A8627, APExBIO), NH4Cl (10001518, SINOPHARM) for 1 h, or RSL3 (B60953133C57E, APExBIO) for 2 h. Cells were harvested by trypsinization and resuspended in 200 µl PBS, then analyzed immediately on a flow cytometer (BD LSRFortessa).

For tumor-infiltrating lymphocyte analysis, tumor of different groups from mice were collected at the end point, ground by grinder and prepared into single cell suspension using 70 µm cell strainers. Single cell suspensions were stained with Zombie NIR Dye (77184, Biolegend), blocked by CD16/CD32 (2.4G2), and then labeled with fluorochrome-conjugated anti-CD45 (30-F11), anti-CD3 (145-2C11), anti-CD4 (GK1.5), anti-CD8a (53-6.7), anti-CD25 (PC61), anti-NK1.1 (PK136), anti-CD11b (M1/70), anti-Ly6G (1A8), anti-Ly6C (HK1.4), anti-F4/80 (T45-2342), anti-CD11c (N418) for 30 min. For transcripts staining, cells were fixed and permeabilized by Transcription Factor Buffer Set (Biosciences, 562574), and stained with anti-FOXP3 (R16-715) for 30 min. For cytokines detection, cells were suspended in complete culture medium and stimulated with Monensin (1: 1000), Ionomycin (1 µg/ml), Brefeldin A (1: 1000) and PMA (20 ng/ml) at 37°C for 4 h in an incubator. Cells were washed, stained with Zombie NIR Dye, and then labeled with anti-CD45, anti-CD3, anti-CD4 and anti-CD8a. Cells were fixed, permeabilized by Fixation Buffer (Biolegend, 420801) and Perm/Wash Buffer (Biolegend, 421002), respectively, and stained with anti-IFNγ (XMG1.2) diluted in Perm/Wash Buffer for 30 min. Finally, cells were resuspended in 500 µL PBS, and assessed immediately on a flow cytometer (BD FACSVerse). The flow cytometry data was analyzed by FlowJo software.

Instrument

BD LSRFortessa, BD verse or Thermo Attune NxT.

Software

FlowJo software version 10

Cell population abundance

No FACS-sorting was involved in the study.

Gating strategy

Tumor cells: The cells were gated on FSC-A/SSC-A basis. To analyze cell death or lipid peroxidaiton, the percentage of PI+ population or mean fluorescence intensity of lipid ROS probe were analyzed. To evaluate the Golgi pH, SEP and mCherry double positive cells were gated and mean fluorescence intensity of SEP and mCherry were analyzed.
Immune cells: The cells were gated on FSC-A/SSC-A basis and single cells were gated on FSC-H/FSC-A basis. Zombie NIR Dye (Biolegend) was used to exclude dead cells. CD8+ T cells: CD45+/CD3+/CD8+/CD4-; CD4+ T cells: CD45+/CD3+/CD4+/CD8-. In CD8 and CD4 gate, the percentage of IFNγ+ cells were analyzed. Treg cells:CD45+/CD3+/CD4+/CD25+/Foxp3+; NK cells:CD45

+/CD3-/NK1.1+; DC cells: CD45+/CD11c+; Macrophage:CD45+/CD11b+/F4-80+;PMN-MDSC:CD45+/CD11b+/Ly6G+/Ly6C low;M-MDSC:CD45+/CD11b+/Ly6G-/Ly6C high.

☒ Tick this box to confirm that a figure exemplifying the gating strategy is provided in the Supplementary Information.
