# Supplementary material for: TMEM87A suppresses ferroptosis and increases cancer immunotherapy resistance by maintaining the Golgi apparatus pH homeostasis
Source: Nat Cancer. 2026 Apr 21;7(5):823–39. doi: 10.1038/s43018-026-01156-9 (PMC13221295; doi:10.1038/s43018-026-01156-9)

Fig 2

Figure 2d

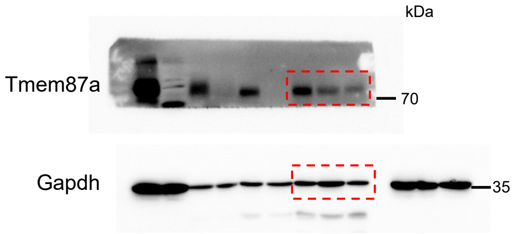

Figure 2f

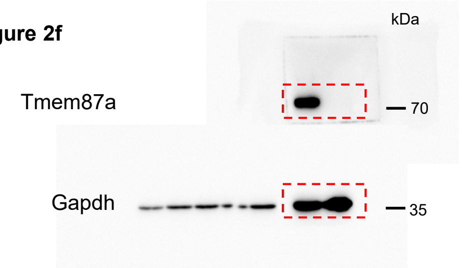

Extended Data Fig 3

Extended Data Fig. 3e

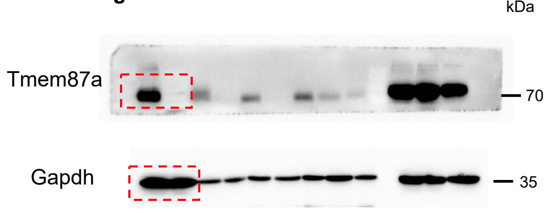

Extended Data Fig. 3f

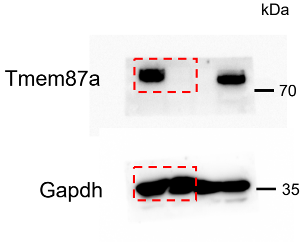

Extended Data Fig. 3k

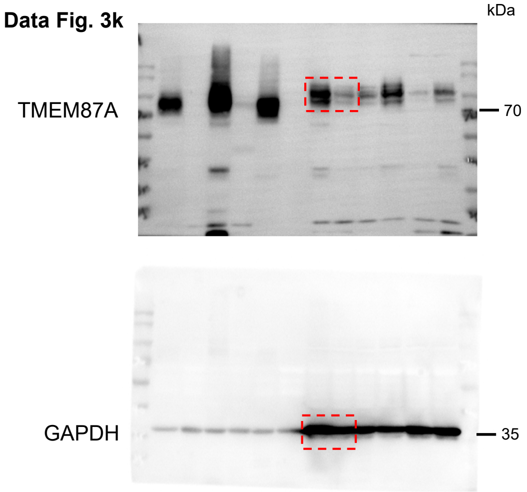

Extended Data Fig. 3l

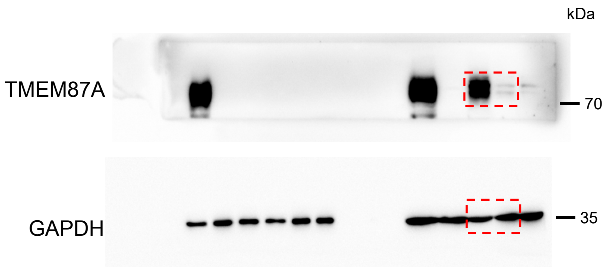

Extended Data Fig. 3o

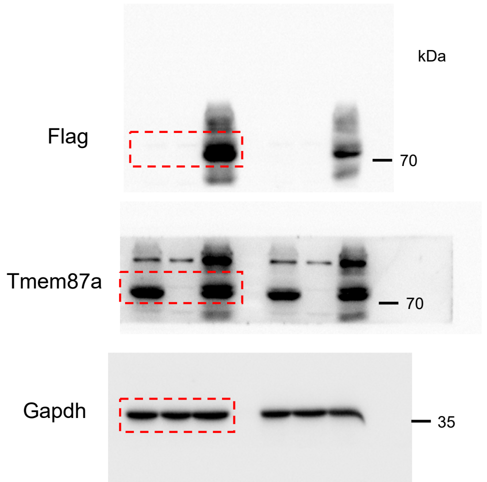

Extended Data Fig. 3p

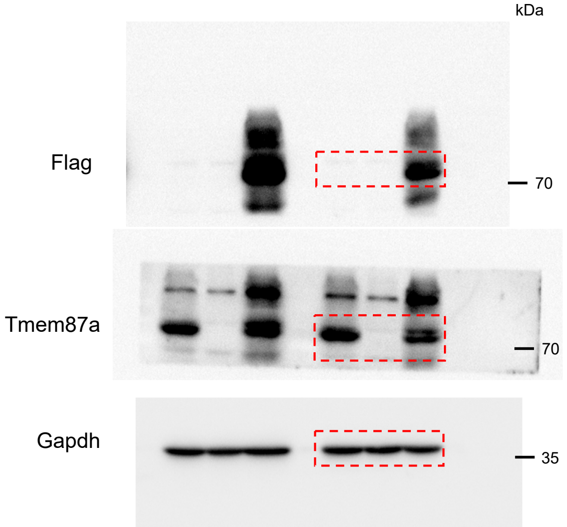

Extended Data Fig. 3q

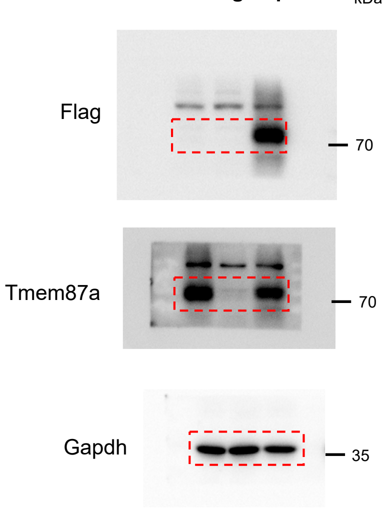

Extended Data Fig 4

Extended Data Fig. 4g

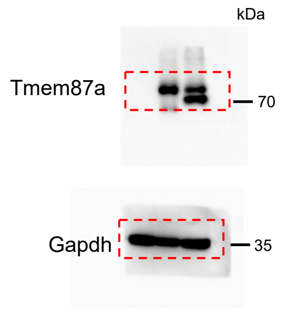

Extended Data Fig 5

Extended Data Fig. 5c

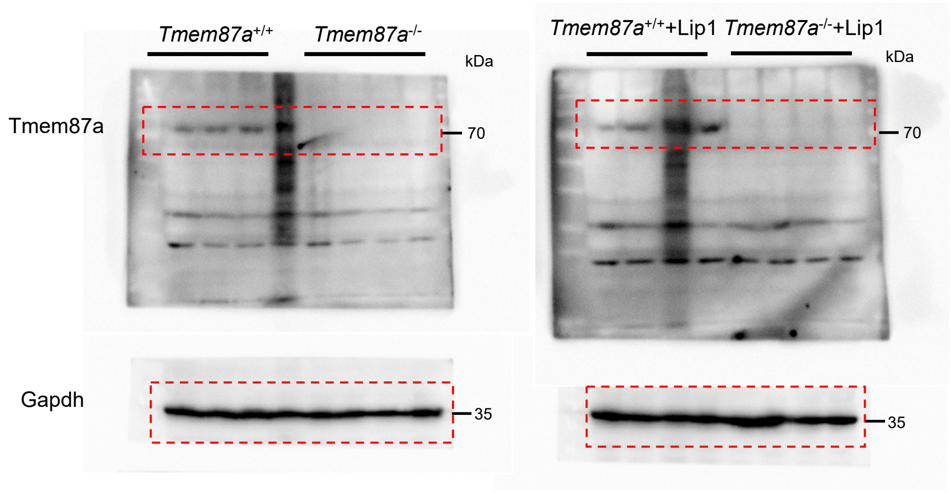

Extended Data Fig. 5j

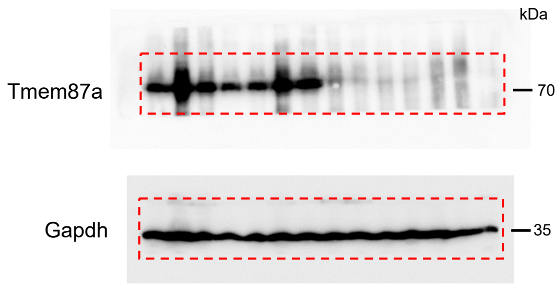

Extended Data Fig 6

Extended Data Fig. 6g

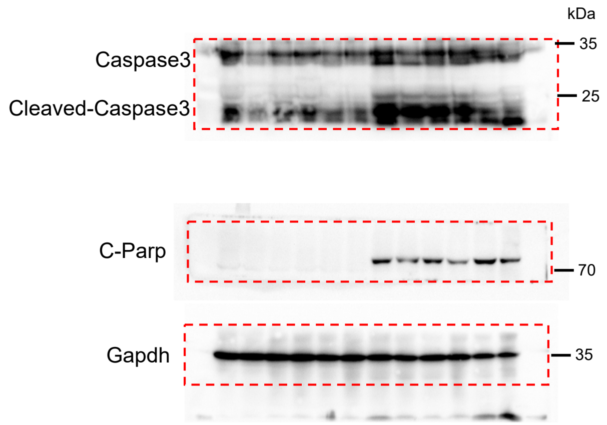

Extended Data Fig 7

Extended Data Fig. 7a

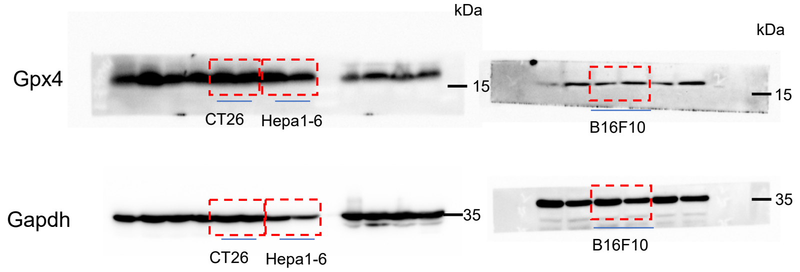

Extended Data Fig. 7b

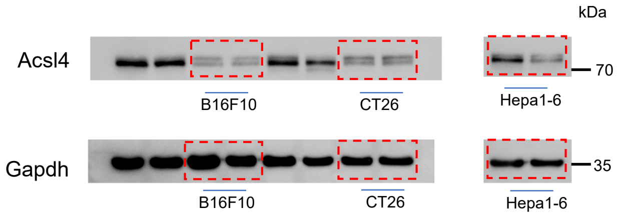

Extended Data Fig. 7c

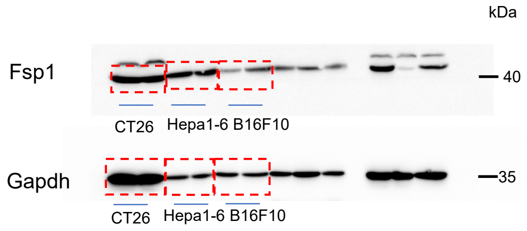

Extended Data Fig. 7g

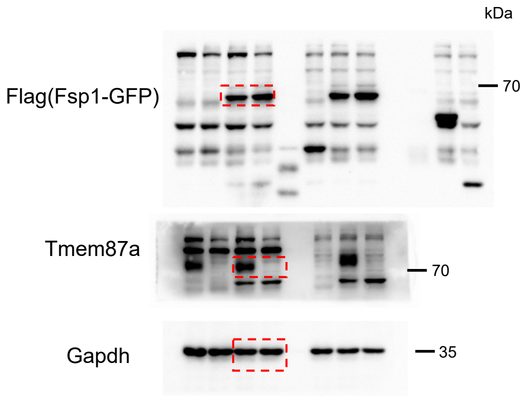

Extended Data Fig. 7h

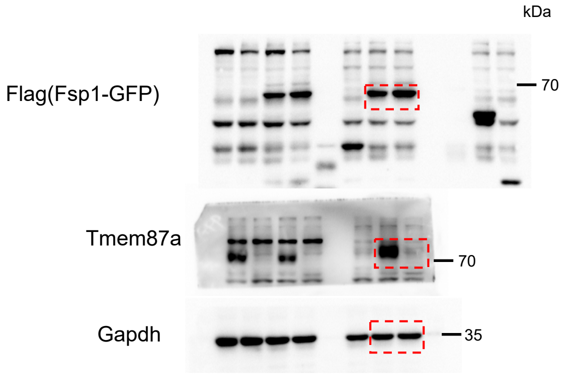

Extended Data Fig. 7l

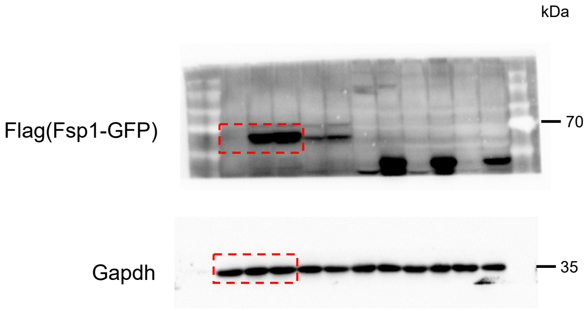

Extended Data Fig. 7m

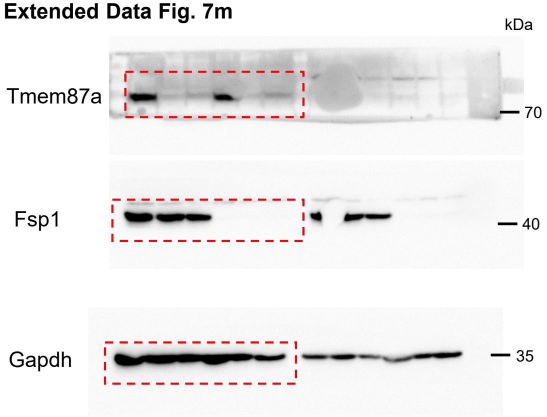

Extended Data Fig. 7n

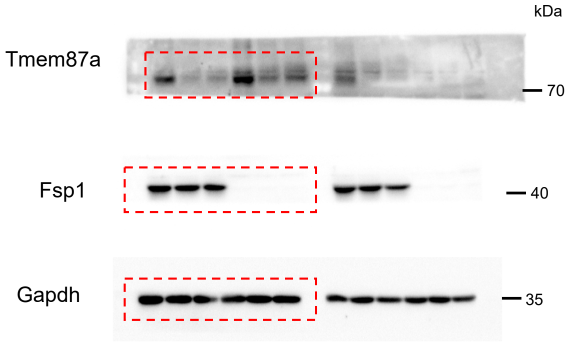

Extended Data Fig 8

Extended Data Fig. 8d

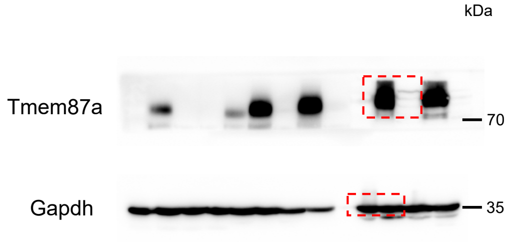

Extended Data Fig. 8h

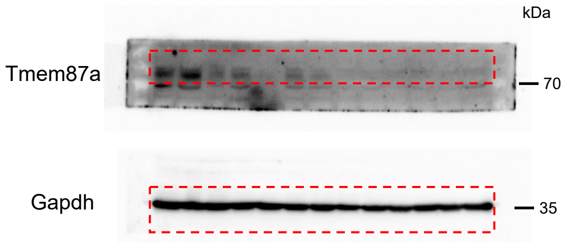

Extended Data Fig. 8i

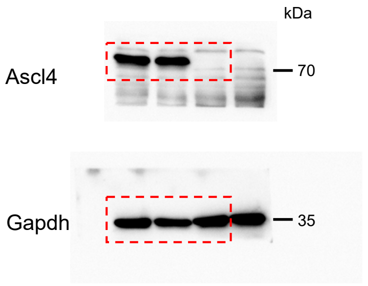

Supplement: Supplementary file 5 — Unprocessed western blots. [file 43018_2026_1156_MOESM5_ESM.pdf]
